# Abscisic acid promotes jasmonic acid biosynthesis via a ‘SAPK10‐bZIP72‐*AOC*’ pathway to synergistically inhibit seed germination in rice (*Oryza sativa*)

**DOI:** 10.1111/nph.16774

**Published:** 2020-07-28

**Authors:** Yifeng Wang, Yuxuan Hou, Jiehua Qiu, Huimei Wang, Shuang Wang, Liqun Tang, Xiaohong Tong, Jian Zhang

**Affiliations:** ^1^ State Key Laboratory of Rice Biology China National Rice Research Institute Hangzhou 311400 China; ^2^ College of Life Science Yangtze University Jingzhou 434025 China

**Keywords:** abscisic acid (ABA), bZIP, jasmonic acid (JA), phosphorylation, rice, seed germination, SnRK2

## Abstract

- Abscisic acid (ABA) and jasmonic acid (JA) both inhibit seed germination, but their interactions during this process remain elusive. Here, we report the identification of a ‘SAPK10‐bZIP72‐*AOC*’ pathway, through which ABA promotes JA biosynthesis to synergistically inhibit rice seed germination.
- Using biochemical interaction and phosphorylation assays, we show that SAPK10 exhibits autophosphorylation activity on the 177^th^ serine, which enables it to phosphorylate bZIP72 majorly on 71^st^ serine. The SAPK10‐dependent phosphorylation enhances bZIP72 protein stability as well as the DNA‐binding ability to the G‐box *cis*‐element of *AOC* promoter, thereby elevating the *AOC* transcription and the endogenous concentration of JA.
- Blocking of JA biosynthesis significantly alleviated the ABA sensitivity on seed germination, suggesting that ABA‐imposed inhibition partially relied on the elevated concentration of JA.
- Our findings shed a novel insight into the molecular networks of ABA–JA synergistic interaction during rice seed germination.

## Introduction

Seeds of cereals such as rice and wheat acquire dormancy during the seed maturation, which enables the offspring to survive from the harsh conditions and initiate the new life cycle after sensing optimal environment cues (Bentsink & Koornneef, 2008). In the agricultural production, seeds acquiring precocious germination are predisposed to cause preharvest sprouting, ultimately leading to losses of grain yield and quality (Gubler *et al*., 2005).

Abscisic acid (ABA) is a primary phytohormone repressing seed germination majorly by inhibiting cell wall loosening and expansion, which is the key step to starting germination (Gimeno‐Gilles *et al*., 2009). The inhibitory effect of ABA on germination has been supported by much genetic evidence. Seeds of typical ABA‐deficient or ABA‐insensitive mutants have faster germination than the wild‐type (WT), while ABA catabolism mutants with high ABA accumulation levels exhibit lower germination rate and speed (Shu *et al*., 2016). About a decade ago, a ‘PYR/PYL/RCAR‐PP2C‐SnRK2’ cascade model for ABA signaling was constituted in *Arabidopsis* (Umezawa *et al*., 2010). In this model, PYR/PYL/RCAR receptors perceive the ABA signal to form a favorable configuration for the binding of PP2C phosphatases, thus releasing their dephosphorylation activity to autophosphorylate SnRK2s. Phosphorylation‐activated SnRK2s further pass the signals to AREB/ABF transcription factors (TFs) through protein phosphorylation, and finally activate the downstream genes to respond to the ABA signals. As the core element in ABA signaling pathway, SnRK2 protein kinases SnRK2.2 and SnRK2.3 have been reported to play essential roles in ABA‐mediated seed germination inhibition, and the *snrk2.2 snrk2.3 snrk2.6* triple mutant became almost completely insensitive to ABA in seed germination in *Arabidopsis* (Fujii & Zhu, 2009). Rice SnRK2 members are termed SAPK1‐10 (osmotic stress/ABA‐activated protein kinase), of which SAPK6, SAPK8, SAPK9 and SAPK10 are reported to be functionally related to ABA signaling, and SAPK10 shows the highest homology to SnRK2.2, SnRK2.3 and SnRK2.6 in Arabidopsis, while its role in seed germination remains unclear (Kobayashi *et al*., 2004; Chae *et al*., 2007; Yuhko *et al*., 2010). To date, only a few ABFs have been identified as SnRK2 substrates, including ABI5 and AtDPBF2 which are implicated in seed maturation and germination in *Arabidopsis* (Lopezmolina *et al*., 2001; Kulik *et al*., 2011). Using a quantitative phosphoproteome strategy, Wang *et al* (2013) identified 58 potential substrate proteins of *Arabidopsis* SnRK2s, but their kinase‐substrate relationships need more verification by biochemical analysis (Wang *et al*., 2013).

Jasmonic acid (JA) and its derivates have been found to inhibit seed germination by disrupting the peroxisomal Adenosine triphosphate (ATP) binding cassette transporter or core β‐oxidation process (Dave *et al*., 2011; Liu *et al*., 2015). Owing to the fact that exogenous ABA or stress‐induced endogenous ABA usually stimulates JA biosynthesis (Adie *et al*., 2007; Avramova, 2017), JA is generally believed to work synergistically with ABA in most of the biological processes, including seed germination (Liu *et al*., 2015; Tang *et al*., 2020), despite the fact that a few exceptional cases in plant biotic stress have also been reported to show antagonistic effects of ABA‐JA (Garcia‐Andrade *et al*., 2011; Xie *et al*., 2018). Previous efforts have provided clues that JAM1, as well as MYC2, may serve as the linkers of ABA and JA signaling (Nakata *et al*., 2013; Aleman *et al*., 2016). Nevertheless, the mechanisms underlying ABA‐mediated JA biosynthesis are still largely elusive, particularly in the seed germination process. In this study, we attempted to address this question by identifying a regulatory pathway ‘SAPK10‐bZIP72‐*AOC*’, through which ABA promotes the JA biosynthesis to synergistically inhibit seed germination. Moreover, we revealed that the inhibitory effect of ABA on seed germination is partially based on the elevated concentration of JA. This study provides a novel insight into the molecular mechanism of ABA and JA synergy in rice seed germination, which would deepen our understanding of the hormone‐mediated fine‐tuning network of seed germination in crops.

## Materials and Methods

### Vector construction and plant transformation

For overexpression of *SAPK10* or *bZIP72*, the coding sequence (CDS) of *SAPK10* or *bZIP72* was amplified using the cDNA of the WT (*Oryza sativa* L. ssp.* japonica* cv Nipponbare) as the template and cloned into the binary vector PU1301 driven by the maize ubiquitin promoter, by using the *Kpn*I and *Bcl*I sites for *SAPK10* and the *Kpn*I and *Spe*I sites for bZIP72. The S177A mutation in *SAPK10* and S71A mutation in *bZIP72* were amplified using overlap PCR (Higuchi *et al*., 1988) and inserted into PU1301 vector to generate PU1301‐*SAPK10^S177A^* or PU1301‐bZIP72*^S71A^*, respectively. Mutants of *SAPK10*, *bZIP72* and *bZIP72*/*TRAB1* were generated by CRISPR/Cas9 system as described previously (Ma *et al*., 2015). Briefly, annealed double‐strand oligos of the gDNA sequences were ligated into the pYLCRISPR/*Cas9*‐MH using *Bsa*I site (Thermo, Waltham, MA, USA). All the binary vectors were transformed into Nipponbare callus using the *Agrobacterium*‐mediated transformation method as described previously (Hiei *et al*., 1994). The primers used were listed in Supporting Information Table S1.

### Seed germination and seedling growth assays

Seed germination assay was performed as previously reported (Lin *et al*., 2015) with a few modifications. Briefly, dehulled rice seeds from WT and transgenic lines in Nipponbare (*O. sativa* L. ssp.* japonica*) background were immersed in 75% ethanol for 1 min. Then the seeds were sterilized in 2.5% (v/v) NaClO for 30 min and subsequently washed five times with sterilized water (each time for 5 min). Rinsed seeds were planted on half‐strength Murashige & Skoog medium (½MS; pH 5.8) with 0.3% plant agar. Then the seeds were placed in a growth chamber (28 ± 2°C, 12 h : 12 h photoperiod with 60% relative humidity) for 4 d to reach the maximum germination rate of the WT seeds under mock treatment. Complete germination was defined as the growth of the coleoptile to 5 mm in length (Lin *et al*., 2015). The seed germination rates were recorded as a percentage every 12 h. Triple biological repeats (each replicate containing 50 seeds) were performed for each sample. Postgermination growth assay was performed as follows: the seedlings were grown using rice nutrient solution as previously described (W. Wang *et al*., 2014; X.F. Wang *et al*., 2014) under different treatments for 10 days, then the shoot height was recorded. The relative germination of the seeds under ABA treatments were determined at 4 d after germination (DAG) and expressed as a percentage of ABA treatment germination rate vs mock treatment germination rate. The sensitivity to ABA treatment was evaluated by analysis of variance and multiple comparisons using R functions ‘aov’ and ‘emmeans’.

### Yeast two‐hybrid assays

Yeast two‐hybrid (Y2H) assays were performed using the matchmaker GAL4 two‐hybrid system (Clontech, CA, USA) according to the manufacturer's instructions. The coding sequences of *SAPK10* were digested with *EcoR*I and *Xho*I and cloned into the pGADT7 vector, the coding sequences of *SAPK10* were digested with *EcoR*I and *Pst*I and cloned into the pGBKT7 vector, and the coding sequences of *bZIP72* were digested with *Nde*I and *EcoR*I and cloned into the pGBKT7 vector, respectively. Primers used were listed in Table S1. The resulting constructs were cotransformed into the yeast strain Y2H Gold (Clontech) and selected on synthetic medium lacking leucine, tryptophan and histidine with 0.04 mg ml^−1^ X‐α‐Gal and 100 ng ml^−1^ Aureobasidin A applied.

### Pull‐down assays

Full‐length CDSs of *bZIP72* and *bZIP72^S71A^* were digested with *EcoR*I and *Sma*I and cloned into the pGEX‐4T‐1 vector (GE Healthcare, Chicago, IL, USA), and the full‐length CDS of *SAPK10* was cloned into pGEX‐4T‐1 using the *EcoR*I and *Sal*I sites, and full‐length CDSs of *SAPK10* and *SAPK10^S177A^* were cloned into pET28a (Thermo), both by using the *EcoR*I and *Xho*I sites. Primers used were listed in Table S1. These recombinant vectors were transformed into *Transetta* (DE3) chemically competent cell (Transgen, Beijing, China), and purified using the glutathione S‐transferase (GST)‐Sefinose™ Kit (Sangon Biotech, Shanghai, China) and 6 × His‐Tagged Protein Purification Kit (CWBIO, Beijing, China) according to the manufacturer's protocols, respectively. The detected interactive proteins were incubated with glutathione high‐capacity magnetic agarose beads (Sigma‐Aldrich) in pull‐down buffer (50 mM Tris‐HCl, pH 7.5, 5% glycerol, 1 mM EDTA, 1 mM DL‐Dithiothreitol (DTT), 1 mM phenylmethylsulfonyl fluoride (PMSF), 0.01% Nonidet P‐40, and 150 mM KCl) at 4°C for 2 h. After washing five times with pull‐down buffer, the beads were suspended in 50 μl 1 × PBS and 10 μl 6 × sodium dodecyl sulfate (SDS) protein loading buffer and boiled for 5 min for immunoblot analysis on 10% SDS‐polyacrylamide gel electrophoresis. Individual bands were detected using Supersignal West Pico Chemiluminescent Substrate (Thermo) and the ChemDoc Touch Imaging system (Bio‐Rad). The dilution for anti‐GST (Yeasen Ltd, Shanghai, China) and anti‐His (Yeasen) was 1 : 5000.

### Coimmunoprecipitation (Co‐IP) assays

The full‐length CDS of *bZIP72* was digested with *Kpn*I and *Spe*I and cloned into the *proUbi*–FLAG vector, and the full‐length CDS of *SAPK10* was digested with *Kpn*I and *Xba*I and cloned into pCAMBIA1300‐35S‐GFP vector, respectively. Primers used were listed in Table S1. bZIP72‐FLAG was transiently coexpressed with empty green fluorescent protein (GFP) or SAPK10‐GFP in tobacco leaves by *Agrobacterium* infiltration. The cotransformed tobacco leaves were then ground into fine powders in liquid nitrogen and resuspended in protein extraction buffer (25 mM Tris‐HCl, pH 7.4, 150 mM NaCl,1 mM EDTA, 1% NonidetP‐40, and 5% glycerol, 1 mM PMSF, 20 μM MG132, and 1× Roche protease inhibitor cocktail (Roche)). After brief centrifugation twice (12 000 ***g*** for 10 min each time), the resulting supernatant was incubated with anti‐FLAG M2 magnetic beads (Sigma‐Aldrich) at 4°C for 2 h. Beads were washed five times with washing buffer (50 mM Tris, pH 7.5, 150 mM NaCl, 0.2% Triton X‐100, 1 mM PMSF, and 1× Roche protease inhibitor cocktail). The immunoprecipitated proteins were suspended in 50 μl of 1 × PBS and 10 μl of 6 × SDS loading buffer, boiled for 5 min, and resolved on 10% acrylamide gels. Individual bands were detected using Supersignal West Pico Chemiluminescent Substrate (Thermo) and the ChemDoc™ Touch Imaging system (Bio‐Rad). The dilution for anti‐FLAG (Sigma‐Aldrich) and anti‐GFP (Yeasen) antibodies was 1 : 5000.

### *In vitro* phosphorylation assays

*In vitro* kinase assays were performed as previously described (Hou *et al*., 2019). Briefly, for the SAPK10 autophosphorylation assay, GST, GST‐SAPK10 or different mutated versions of GST‐SAPK10 (GST‐SAPK10^S177A^, GST‐SAPK10^T178A^ and GST‐SAPK10^S177A/T178A^) was expressed in *Transetta* (DE3) chemically competent cell (Transgen), and purified using the GST‐Sefinose™ Kit (Sangon Biotech, Shanghai, China) according to the manufacturer’s instructions, respectively. The purified proteins (100 ng) were incubated with 1 µg calf intestinal phosphatase (CIAP; Takara, Dalian, China) at 37°C for 30 min, and then separated by electrophoresis on 10% acrylamide gels. Phosphorylated bands were detected using biotinylated Phos‐tag™ zinc complex BTL111 purchased from Wako (Osaka, Japan) according to the manufacturer’s instructions. For the kinase assay of SAPK10 and bZIP72, GST‐bZIP72 or GST‐bZIP72^S71A^ was coexpressed with His‐SAPK10 in *Transetta* (DE3) chemically competent cell, and purified using the GST‐Sefinose™ Kit (Sangon Biotech) and 6 × His‐Tagged Protein Purification Kit (CWBIO), respectively. The detection of bZIP72 phosphorylated bands was the same as that of SAPK10 autophosphorylation bands.

### *In vivo* phosphorylation assays

The experiments were performed as described previously (Zhou *et al*., 2018) with a few modifications. *SAPK10‐FLAG* and *bZIP72‐FLAG* overexpression lines were hydroponically cultured for 2 wk, then transferred to the solutions containing different concentrations of ABA (0, 25, 50, 100 µM) for 6 h, respectively. Then the seedlings of *SAPK10‐FLAG* or *bZIP72‐FLAG* were harvested and ground into fine powders in liquid nitrogen and resuspended in protein extraction buffer (25 mM Tris‐HCl, pH 7.4, 150 mM NaCl, 1 mM EDTA, 1% NonidetP‐40, and 5% glycerol, 1 mM PMSF, 20 μM MG132, and 1 × Roche protease inhibitor cocktail (Roche). After brief centrifugation twice (12 000 ***g*** for 10 min each time), the resulting supernatant was incubated with anti‐FLAG M2 magnetic beads (Sigma‐Aldrich) at 4°C for 1 h. The beads were washed five times with washing buffer (50 mM Tris, pH 7.5, 150 mM NaCl, 0.2 % Triton X‐100, 1 mM PMSF, and 1 × Roche protease inhibitor cocktail). Bound proteins were eluted with 50 μl of 1 × PBS and 10 μl of 6 × SDS loading buffer, boiled for 5 min, and resolved on 10% acrylamide gels. The serine phosphorylation of SAPK10‐FLAG and bZIP72‐FLAG were detected using antiphosphoserine antibody. The dilution for antiphosphoserine antibody (Abcam, Cambridge, UK) and anti‐FLAG (Sigma‐Aldrich) antibodies was 1 : 5000.

### Cell‐free degradation assays

The experiments were conducted as described previously (Lv *et al*., 2014). Briefly, the wild‐type (*japonica* cv Nipponbare) were hydroponically cultured under mock or +ABA (2 µM ABA) nutrition solutions for 2 wk. Then the seedlings were harvested and ground into fine powders in liquid nitrogen, then resuspended in the degradation buffer (25 mM Tris‐HCl, pH 7.5, 10 mM NaCl, 10 mM MgCl_2_, 4 mM PMSF, 5 mM DTT and 10 mM ATP). After brief centrifugation twice (12 000 ***g*** for 10 min each time), the resulting total proteins (30 µg of each) were incubated with recombinant GST‐bZIP72 or GST‐bZIP72^S71D^ (100 ng of each) at 28°C for the individual assays. The reactions were taken at indicated intervals for determination of bZIP72 abundance by immunoblotting. The protein intensities were quantified using imagej software. The dissociation‐one phase exponential decay curve was plotted on a semilog graph using graphpad prism (5.0) software to calculate the half‐life of GST‐bZIP72. The dilution for anti‐GST (Yeasen) was 1 : 5000.

### Electrophoresis mobility shift assay (EMSA)

Recombinant p‐GST‐bZIP72 (phosphorylated GST‐bZIP72) was purified from *Transetta* (DE3) chemically competent cell (Transgen) coexpressing GST‐ bZIP72 and His‐SAPK10. The EMSA probes in a length of 59 nt in the *AOC* promoter were commercially synthesized by Tsingke Co. (Tsingke Biotech, Hangzhou, China) and labeled using an EMSA Probe Biotin Labeling Kit (Beyotime, Shanghai, China) according to the manufacturer’s instructions. Probes used were listed in Table S1. An equal amount of purified recombinant proteins were preincubated with EMSA/gel‐shift binding buffer (Beyotime) at 25°C for 20 min, then incubated with 20 fmol labeled probes with or without nonlabeled competitive DNA probes for another 20 min. Then the incubated mixed samples were separated by electrophoresis on 6% acrylamide gels and transferred to the Nylon membrane (Beyotime, Shanghai, China). The labeled DNA probes was detected using the LightShift Chemiluminescent EMSA kit (Thermo) according to the manufacturer’s instructions. Migration of biotin‐labeled probes was detected using Supersignal West Pico Chemiluminescent Substrate (Thermo) and the ChemDoc™ Touch Imaging system (Bio‐Rad).

### Chromatin immunoprecipitation‐quantitative PCR (ChIP‐qPCR)

The experiments were conducted as described previously (Hou *et al*., 2015). Briefly, chromatin was isolated from 2 g crosslinked leaves of the wild‐type plants. Isolated chromatin was sonicated for DNA fragmentation ranging from 200 to 700 bp. Subsequently, the DNA/protein complex was immune‐precipitated with polyclonal rabbit bZIP72 antibody against the amino acid residues 258 to 271 (C‐ DSGDKGNSDLSSPT ‐COOH) of bZIP72, which was commercially synthesized and affinity‐purified by Genescript company (Genescript, Shanghai, China). Then the immunoprecipitated DNA was purified with phenol/chloroform after reverse cross‐linking and proteinase K treatment. The immunoprecipitated and input DNA were used for quantitative reverse transcription polymerase chain reaction (qRT‐PCR) with gene‐specific primers, respectively. The gene‐specific primers used were listed in Table S1. The quantitative PCR results were analyzed according to the manual of the Magna ChIP™ HiSens kit (Millipore, Darmstadt, Germany). The quantitative ChIP‐qPCR was performed with triple biological replicates.

### Luciferase transient transcriptional activity assay

The CDS of *bZIP72* and *bZIP72^S71A^* were cloned into ‘None’ vectors as effectors by using the *BamH*I and *EcoR*I sites, and the promoter region of *AOC* was cloned into 190fLUC vector as reporter by using the *Hind*III and *Bgl*II sites. The primers used were listed in Table S1. Then the plasmids were transformed into rice protoplasts as reported previously (Xie & Yang, 2013). Then the transformed protoplasts were resuspended in 50 μl lysis buffer, and 30–50 μl lysate was used to measure the luciferase activity. Firefly luciferase assay substrate buffer (100 μl) was added into the lysate and the firefly luciferase (fLUC) activity was measured. Subsequently, Stop & Renilla luciferase substrate buffer (100 μl) was added to the reaction and the Renilla luciferase (rLUC) activity was determined. All the luciferase activities were detected by the Dual Luciferase Reporter Gene Assay Kit (Beyotime) using the Tecan infinite M200 system (Tecan, Mannedorf, Switzerland). The relative luciferase activity was calculated as the ratio between fLUC and rLUC (fLUC/rLUC). AtUbi3:rLUC was used as an internal control. The luciferase activity for each sample was detected with triple biological replicates.

### Quantification of JA concentrations in rice

Jasmonic acid extraction and quantification were performed as described previously (Zhang *et al*., 2018). Briefly, *c*. 0.1 g seedlings grown on ½MS medium for 4 d were ground into fine powers and extracted with 1 ml plant hormone extraction buffer (isopropanol : H_2_O : hydrochloric acid, 2 : 1 : 0.002 v/v/v). The extract was gently agitated at 4°C for 30 min. Then 20 ml of dichloromethane was added to the samples and gently agitated at 4°C for 30 min. Subsequently, the samples were centrifuged at 8000 ***g*** for 10 min and the organic phase was extracted and dried with liquid nitrogen. Ultimately, the pellets were dissolved in 150 ml of 0.1% methanol and filtered with 0.22 µm filter membranes. The JA contents of the purified products were quantified using high‐performance liquid chromatography‐tandem MS (HPLC‐MS/MS). The MS conditions were performed as reported previously (Zhang *et al*., 2018).

## Results

### SAPK10 is autophosphorylated on serine 177

**Fig. 1:**
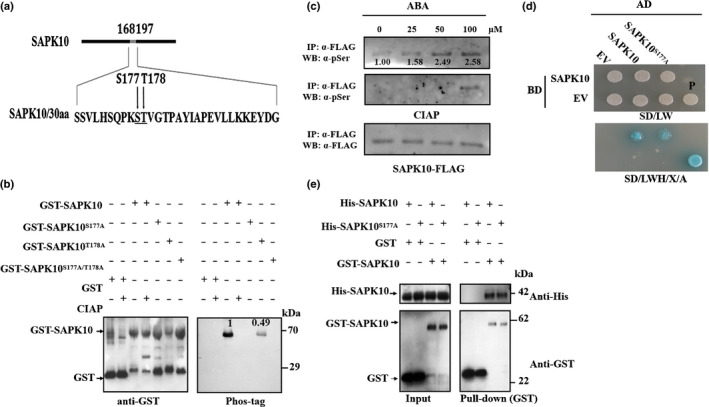
SAPK10 is autophosphorylated on serine 177. (a) Schematic representation of the putative autophosphorylation sites of SAPK10. Ser‐177 and Thr‐178 in the T‐ loop region of SAPK10 (amino acids 168–197) are indicated in bold. (b) Serine 177 is the key phosphosite of SAPK10. Equal amounts of GST‐SAPK10 and other mutated versions (GST‐SAPK10^S177A^, GST‐SAPK10^T178A^, GST‐SAPK10^S177A/T178A^) were detected with anti‐GST antibody (left panel) and the phosphorylated proteins were detected with biotinylated Phos‐tag™ zinc BTL111 complex (right panel). The GST protein was used as negative control. GST, glutathione S‐transferase. (c) SAPK10 phosphorylation was enhanced under abscisic acid (ABA) treatment. Two‐week‐old *pro35S:SAPK10‐FLAG* seedlings (Nipponbare, *Oryza sativa* ssp*. japonica*) were treated with ABA in different concentrations (0, 25, 50 and 100 μM) for 6 h. Equal amounts of immunoprecipitated SAPK10‐FLAG proteins were immunoblotted against antiphosphoserine (a‐pSer) antibody (top panel), treated with calf intestinal phosphatase (CIAP) at 37°C for 30 min and against a‐pSer antibody (middle panel) and anti‐FLAG antibody (bottom panel). The relative intensity of SAPK10‐FLAG under no‐ABA treatment was set to 1.00. (d) Yeast two‐hybrid assay. Yeast cells cotransformed with SAPK10 or SAPK10^S177A^ fused to the GAL4 activation domain (SAPK10‐AD or SAPK10^S177A^‐AD) and SAPK10 fused to the GAL4‐binding domain (SAPK10‐BD) were grown on selective media. BD, pGBKT7; AD, pGADT7; EV, empty vector; SD/LW, ‐Leu‐Trp; SD/LWH, ‐Leu‐Trp‐His; P, positive control using pGADT7‐T + pGBKT7‐53; X: X‐α‐Gal in 0.04 mg ml^−1^; a, Aureobasidin A in 100 ng mI^−1^. (e) Pull‐down assay. Purified His‐SAPK10, His‐SAPK10^S177A^, GST and GST‐SAPK10 were subjected to pull‐down assays and detected with anti‐His and anti‐GST antibodies, respectively. Molecular mass markers are shown (kDa).

As the closest ortholog of OST1 in *Arabidopsis* and AAPK in *Vicia faba*, SAPK10, which is an ABA‐inducible SnRK2 type kinase in rice, has been reported to be a core component in ABA signaling (Kobayashi *et al*., 2004, 2005). It is demonstrated that the function of SnRK2s, such as OST1, is activated by autophosphorylation within the activation T‐loop domain (Belin *et al*., 2006; Ng *et al*., 2011). Sequence similarity analysis of SAPK10 identified two putative phosphorylation sites, Serine 177 and Threonine 178 (S177 and T178, respectively) within the T‐loop region, which were conserved with those of OST1 in *Arabidopsis* (Figs 1a, S1). Autophosphorylation assay of SAPK10 showed that a phosphorylation band of purified GST‐SAPK10 was detected by phos‐tag, and it disappeared after CIAP treatment, while GST as a negative control was not phosphorylated, indicating that SAPK10 has an autophosphorylation activity on its own (Fig. 1b). When Ser177 and Thr178 of SAPK10 were replaced by alanines (SAPK10^S177A^ and SAPK10^T178A^, respectively), the autophosphorylation band was not detected in SAPK10^S177A^ and SAPK10^S177A/T178A^ (Fig. 1b). However, the phosphorylation intensity of SAPK10^T178A^ was also decreased to nearly half that of the native SAPK10 (Fig. 1b). Therefore, Ser177 might be the major autophosphorylation site of SAPK10. Moreover, we treated proUbi:SAPK10‐FLAG seedlings with exogenous ABA at various concentrations, and purified the SAPK10‐FLAG proteins to examine their phosphorylation intensities by immunoblot. Obviously, the application of ABA significantly increased the SAPK10 phosphorylation intensity in a dosage‐dependent manner, and the phosphorylation bands were effectively removed by CIAP treatment, suggesting that the autophosphorylation on SAPK10 is induced by the ABA concentration *in vivo* (Fig. 1c). The autophosphorylation activity of SAPK10 intrigued us to test the protein self‐binding ability. The Y2H and *in vitro* pull‐down assays clearly demonstrated that SAPK10 could physically bind itself to form a homodimer. The autophosphorylation on SAPK10 may not affect its protein‐binding ability, as SAPK10 was found to physically interact with SAPK10^S177A^ in yeast and *in vitro* (Fig. 1d–e).

### Overexpression of *SAPK10* confers rice with hypersensitivity to ABA

**Fig. 2:**
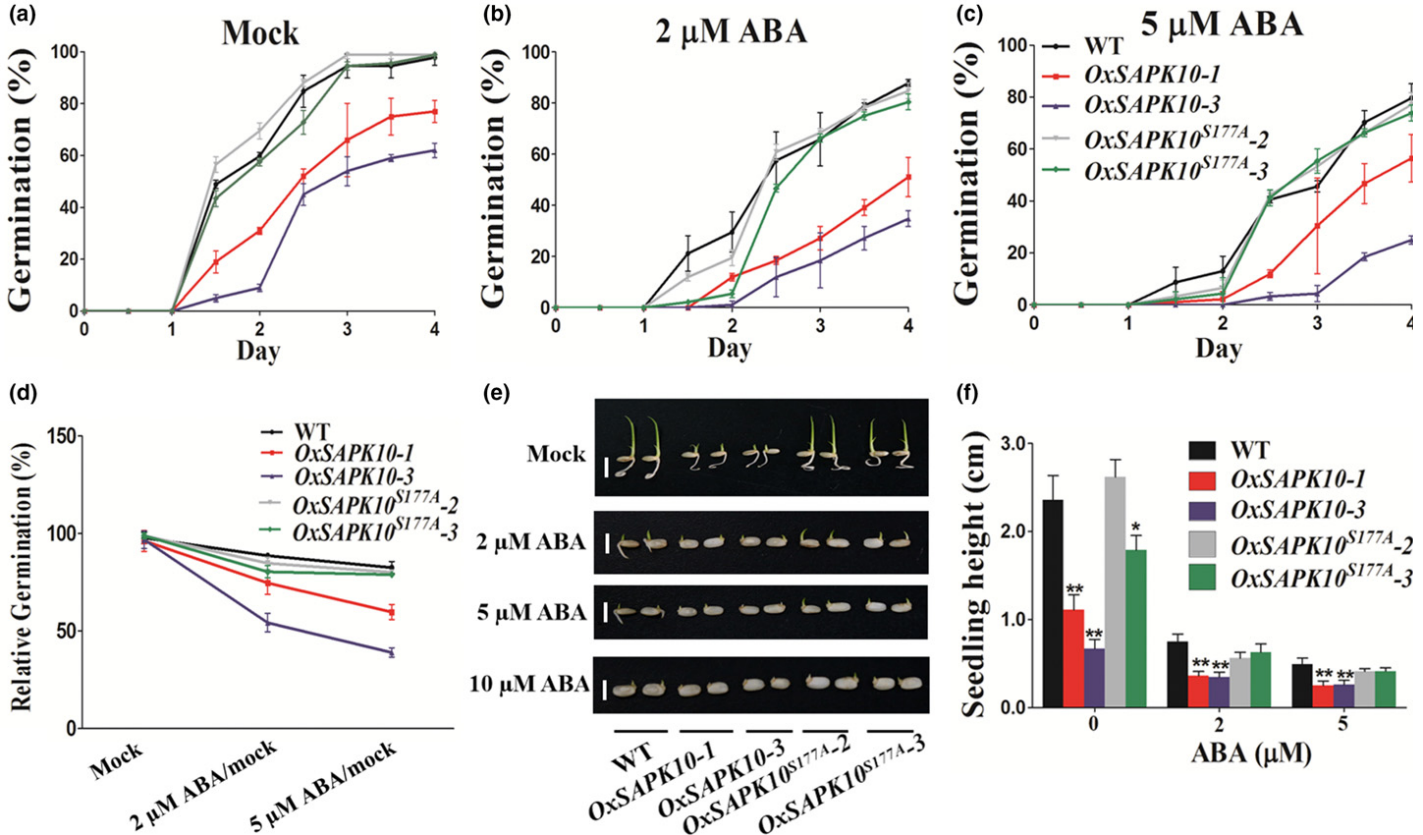
Overexpression of *SAPK10* confers rice with hypersensitivity to abscisic acid (ABA). (a) Germination time courses of wild‐type (WT), *OxSAPK10* and *OxSAPK10^S177A^* seeds grown on half‐strength Murashige & Skoog (½MS) medium containing different concentrations of ABA (0, 2 and 5 μM), respectively. (b) Relative germination of the WT, *OxSAPK10* and *OxSAPK10^S177A^* seeds under ABA treatments were determined after 4 d and expressed as a percentage of those grown under the ‘mock’ condition. (c) Germination phenotypes of the WT, *OxSAPK10* and *OxSAPK10^S177A^* treated with 0, 2 and 5 μM ABA, respectively. Photographs were taken on day 7. Bars, 1cm. (d) Seedling heights of the WT, *OxSAPK10* and *OxSAPK10^S177A^* in accordance with (c). Error bars indicate SD with biological triplicates (*n* = 3, each replicates containing 50 seeds) in (a) and 50 biological replicates (*n* = 50) in (d). Asterisks indicate the significance of differences between the WT and transgenic lines as determined by Student's *t*‐test analysis: *, *P* < 0.05; **, *P* < 0.01.

*SAPK10* overexpression lines (*OxSAPK10*) as well as CRISPR/Cas9‐mediated knockout mutants (*crsapk10*) were generated to dissect the biological roles of SAPK10 in rice. The seeds of T_2_ homozygous *crsapk10* lines, which harbored an A insertion or an A/G insertion in the first exon and shifted the open reading frame of *SAPK10*, showed similar germination rate as the WT under the conditions with or without the additions of ABA (Fig. S2). This may be ascribed to the functional redundancy of *SAPK10* with other ABA‐responsive *SAPK* members like *SAPK6*,* SAPK8* and *SAPK9*, as the transcription levels of *SAPK8* and *SAPK9* were elevated when *SAPK10* was knocked out (Fig. S3). Further developing higher‐order mutants of *SAPK6*,* 8* and *9* with *SAPK10* would be helpful to clarify this issue more clearly. Two representative *OxSAPK10* lines with substantially increased transcriptional level were also picked for seed germination assays (Fig. S4). Under no‐ABA conditions, the seed germination and seedling growth of *OxSAPK10* lines were significantly retarded in comparison with the WT (Fig. 2a–c). Similarly, *OxSAPK10* displayed more severe ABA‐mediated repression on seed germination and seedling growth than did the WT (Fig. 2a–d). To evaluate the relative ABA sensitivity of the seeds, we compared the germination rates at 4 DAG under mock and ABA treatments. As a result, the WT relative germination rates of 2 μM ABA/mock and 5 μM ABA/mock were 93% and 84%, respectively. However, for the *OxSAPK10* seeds, these numbers decreased to 61% and 56%, respectively. Thus, *OxSAPK10* is hypersensitive to ABA when compared with the WT (*P* < 0.01) (Fig. 2b). Subsequently, *SAPK10^S177A^* overexpression lines (*OxSAPK10^S177A^*) were generated to explore the effects of autophosphorylation of SAPK10 on its own biological functions (Fig. S4). The lines *OxSAPK10^S177A^‐2* and *OxSAPK10^S177A^‐3*, which have comparable *SAPK10* transcriptional level with *OxSAPK10‐3*, was selected for phenotyping (Fig. S4). Interestingly, in comparison with the delayed seed germination and seedling growth in *OxSAPK10‐3*, the *OxSAPK10^S177A^* line lost its hypersensitivity to ABA, and showed similar germination rate and seedling growth as that of the WT (Fig. 2). Thus, these results indicated that blocking of the autophosphorylation site impairs the SAPK10 function in ABA signaling and inhibiting seed germination.

### SAPK10 phosphorylates and stabilizes bZIP72

**Fig. 3:**
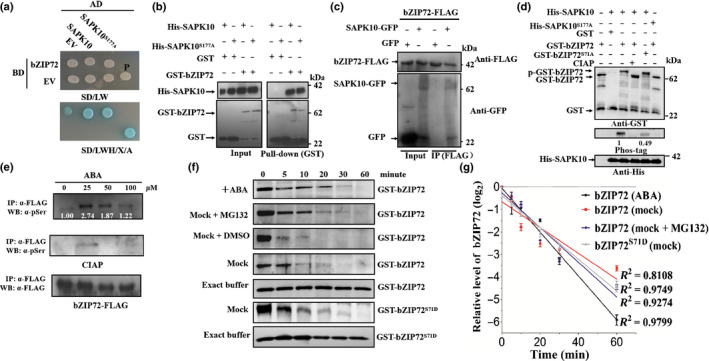
SAPK10 phosphorylates and stabilizes bZIP72. (a) Yeast two‐hybrid assays. Yeast cells cotransformed with SAPK10‐AD or SAPK10^S177A^‐AD and bZIP72 fused to the GAL4‐binding domain (bZIP72‐BD) were grown on selective media. BD, pGBKT7; AD, pGADT7; EV, empty vector; SD/LW, ‐Leu‐Trp; SD/LWH, ‐Leu‐Trp‐His; P, positive control using pGADT7‐T + pGBKT7‐53; X: X‐α‐Gal in 0.04 mg ml^−1^. (a) Aureobasidin A in 100 ng ml^−1^. (b) Pull‐down assay. Purified His‐SAPK10, His‐SAPK10^S177A^, GST and GST‐bZIP72 were subjected to pull‐down assays and detected with anti‐His and anti‐GST antibodies, respectively. (c) Coimmunoprecipitation (Co‐IP) assay. GFP, SAPK10‐GFP and bZIP72‐FLAG extracted from infiltrated tobacco (*Nicotiana benthamiana*) leaves were used in a Co‐IP assay. Precipitates were detected with anti‐GFP and anti‐FLAG antibodies, respectively. (d) bZIP72 was phosphorylated by SAPK10 at Ser71. Equal amounts of the recombinant proteins were detected with anti‐GST (top panel) and anti‐His (bottom panel) antibodies, respectively. The phosphorylated proteins were detected with biotinylated Phos‐tag™ zinc BTL111 complex (middle panel). GST‐bZIP72 protein alone or GST purified from coexpressed forms with His‐SAPK10 was used as the negative control. p‐GST‐bZIP72, GST‐bZIP72 phosphorylated band. The relative intensity of GST‐bZIP72 phosphorylated band was set to 1.00 in the middle panel. (e) *In vivo* phosphorylation of bZIP72 was enhanced under abscisic acid (ABA) treatment. Two‐week‐old *pro35S:bZIP72‐FLAG* seedlings (Nipponbare, *Oryza sativa* ssp.* japonica*) were treated with ABA at different concentrations (0, 25, 50 and 100 μM) for 6 h. Equal amount of immunoprecipitated bZIP72‐FLAG proteins were immunoblotted against antiphosphoserine (α‐pSer) antibody (top panel), treated with calf intestinal phosphatase (CIAP) at 37°C for 30 min and against a‐pSer antibody (middle panel), and anti‐FLAG antibody (bottom panel). The relative intensity of bZIP72‐FLAG phosphorylated band under no‐ABA treatment was set to 1.00 in the top panel. (f) Cell‐free degradation assay of GST‐bZIP72 in the absence or presence of ABA. Equal amounts of total plant (Nipponbare) proteins (30 μg) were incubated with GST‐bZIP72 or GST‐bZIP72^S71D^ (mimicking the phosphorylation form of bZIP72) and detected with anti‐GST antibody. For control, 100 ng GST‐bZIP72 or GST‐bZIP72^S71D^ was incubated with 30 μl of extract buffer. (g) The relative remaining amount of bZIP72 protein was calculated and plotted on a semilog graph in accordance with (f). A ratio of protein signal at the corresponding time to the signal at the start (0 min) was shown as a dissociation‐one phase exponential decay curve. Error bars indicate SD with biological triplicates (*n* = 3).

After screening over two million colonies from a rice seed‐derived cDNA library using Y2H system, we found that transcription factor bZIP72 could interact with SAPK10. Interestingly, *bZIP72* and *SAPK10* both have relatively high transcriptional levels in developing seeds and could be significantly induced by exogenous ABA during seed germination (Fig. S5a,b). Their expression patterns in other various tissues and growth stages, as well as their subcellular localizations, are largely overlapped (Fig. S5c–e). The interaction between SAPK10 and bZIP72 was further validated by Y2H, *in vitro* pull‐down and *in vivo* Co‐IP assays, though bZIP72 somehow displayed weak autoactivation activity in Y2H (Fig. 3a–c). In support of our conclusion that autophosphorylation on SAPK10 does not affect its protein–protein binding ability, SAPK10^S177A^ could also physically bind with bZIP72 in yeast and *in vitro*, just as SAPK10 did (Fig. 3a,b). Meanwhile, almost equal amounts of the His‐SAPK10 and His‐SAPK10^S177A^ proteins were coimmunoprecipitated by GST‐bZIP72 when the pull‐down assays were performed in parallel (Fig. 3b), suggesting that ABA‐induced phosphorylation on SAPK10 has little or no impact on its binding intensity to bZIP72 either.

Given the protein kinase feature of SAPK10, the kinase‐substrate relationship between SAPK10 and bZIP72 was tested by kinase assay in *E.coli*. As shown in Fig. 3(d), the GST‐bZIP72 was only phosphorylated by His‐SAPK10, but not by SAPK10^S177A^, indicating that the autophosphorylation on SAPK10 is a key switch controlling its kinase activity (Fig. 3d). Our previous phosphoproteomic study of ABA‐induced protein phosphorylation revealed that exogenous ABA enhanced the bZIP72 phosphorylation intensity on Serine 71 (Qiu *et al*., 2017). Thus, we attempted to specify the residues responsible for SAPK10‐mediated phosphorylation on bZIP72 by using bZIP72^S71A^ as the substrate for the kinase assay. The phosphorylation intensity of bZIP72^S71A^ is only half that of the native bZIP72, suggesting that Ser71 is a major site of the SAPK10‐mediated phosphorylation (Fig. 3d). Moreover, we found that the *in vivo* phosphorylation intensity on bZIP72 also displayed an ABA dosage‐dependent pattern, which is quite a similar pattern to SAPK10 (Fig. 3e).

Protein phosphorylation has been linked to targeting protein turnover and stability (Chen *et al*., 2015). Therefore, we attempted to check the influences of SAPK10‐mediated phosphorylation on the stability of bZIP7 by cell‐free protein degradation assay. As shown in Fig. 3(f) and (g), treatment of the seedlings with ABA significantly extended the half‐life of GST‐bZIP72 to 10 min, whereas GST‐bZIP72 was degraded to half amount in only 6 min when the mock protein extracts were used, suggesting that ABA enhanced the protein stability of bZIP72 (Fig. 3f,g). On the other hand, by using GST‐bZIP72^S71D^ as the substrate, in which the Ser71 was replaced by aspartic acid to mimic constitutive phosphorylation status of bZIP72, we found that the mimicked phosphorylation significantly stabilized the protein with a half‐life of 9 min when incubated with the mock total protein extracts (Fig. 3f‐g). The addition of MG132, a 26S proteasome inhibitor, drastically delayed the *in vitro* degradation of GST‐bZIP72, which implied that the protein is under the degradation of the Ubiquitin/26S proteasome pathway (Fig. 3f‐g). Taken together, these results strongly suggested that the stability of bZIP72 was enhanced by ABA and SAPK10‐mediated phosphorylation on Ser71.

### bZIP72 and TRAB1 functions redundantly in ABA signaling

**Fig. 4:**
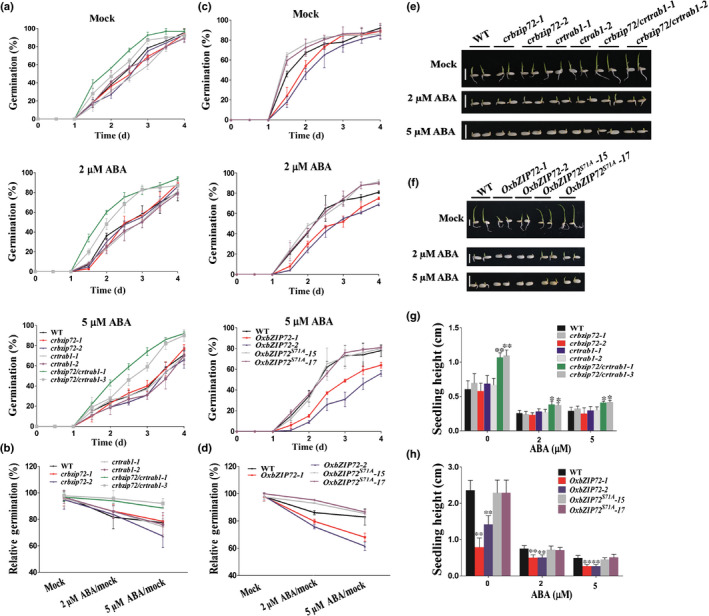
*bZIP72* and *TRAB1* function redundantly in abscisic acid (ABA) signaling in Nipponbare. (a) Germination time courses of the wild‐type (WT), *crbzip72*, *crtrab1* and *crbzip72/crtrab1* seeds grown on half‐strength Murashige & Skoog (½MS) medium containing different concentrations of ABA (0, 2 and 5 μM). (b) The relative germination of the WT, *crbzip72*, *crtrab1* and *crbzip72/crtrab1* seeds under ABA treatments were determined after 4 d and expressed as a percentage of those grown under ‘mock’ conditions. (c) Germination time courses of the WT, *OxbZIP72* and *OxbZIP72^S71A^* seeds grown on ½MS medium containing different concentrations of ABA (0, 2 and 5 μM). (d) The relative germination of the WT, *OxbZIP72* and *OxbZIP72^S71A^* seeds under ABA treatments were determined after 4 d and expressed as a percentage of those grown under ‘mock’ conditions. (e) Germination phenotypes of the WT, *crbzip72*, *crtrab1* and *crbzip72/crtrab1* treated with 0, 2 and 5 μM ABA. Photographs were taken on day 4. Bars, 1 cm. (f) Germination phenotypes of the WT, *OxbZIP72* and *OxbZIP72^S71A^* treated with 0, 2 and 5 μM ABA. Photographs were taken on day 7. Bars, 1cm. (g) Seedling heights of the WT, *crbzip72*, *crtrab1* and *crbzip72/crtrab1* in accordance with (e). (h) Seedling heights of the WT, *OxbZIP72* and *OxbZIP72^S71A^* in accordance with (f). (a, c, g, h) Error bars indicate SD with biological triplicates (*n* = 3, each replicate containing 50 seeds) (a, c) and 50 biological replicates (*n* = 50) (g, h). Asterisks indicate the significance of differences between the WT and transgenic lines as determined by Student's *t*‐test analysis: *, *P* < 0.05; **, *P* < 0.01.

The kinase‐substrate relationship between SAPK10 and bZIP72 motivated us to investigate the function of bZIP72 in ABA signaling by generating various types of mutants and overexpression lines. Interestingly, the CRISPR/Cas9‐mediated *bzip72* homozygous mutants (*crbzip72*) did not show any significant differences with the WT in seed germination, although we have confirmed the indel mutations in *bZIP72* (Figs 4a,b,e,g, S6a,b). It was assumed that the loss‐of‐function of *bZIP72* was compensated by its closest homolog, *TRAB1/OsbZIP66* (*LOC_Os08g36790*), because transcription of *TRAB1* was significantly upregulated when *bZIP72* was mutated (Fig. S7). Subsequently, we knocked out both genes simultaneously (Fig. S6c,d), and found that the *bzip72/trab1* double mutants *(crbzip72/crtrab1)* became less sensitive to ABA during seed germination, while the *trab1* single mutants (*crtrab1*) were very similar to the WT and *bzip72* in phenotype (Fig. 4a,b,e,g). The result suggested that *bZIP72* and *TRAB1* functions redundantly in ABA signaling. Despite the reduced sensitivity of *bzip72/trab1* in seed germination, it should be noted that the seedling growth of *bzip72/trab1* and the WT did not show significant differences (*P* > 0.05), implying that ABA‐mediated repression on germination and shoot growth may depend on different pathways or mechanisms. Despite the reduced sensitivity of *bzip72/trab1* in seed germination, it should be noted that the seedling growth of *bzip72/trab1* and the WT did not show significant differences (*P* > 0.05), implying that ABA‐mediated repression on germination and shoot growth may depend on different pathways or mechanisms. Similar to the phenotypes observed in *OxSAPK10*, *bZIP72* overexpression lines showed retarded seed germination and seedling growth under no‐ABA application, and enhanced sensitivity to ABA treatment, as 2 μM ABA/mock and 5 μM ABA/mock relative germination rates were 79% and 66% for *OxbZIP72*, respectively, and 88% and 85% for the WT (Figs 4c,d,f,g, S8). To further investigate the effect of SAPK10‐mediated phosphorylation on the biological function of bZIP72, the lines *OxbZIP^S71A^‐15* and *OxbZIP72^S71A^‐17*, which have comparable *bZIP72* transcriptional level with *OxbZIP72*, were selected for phenotyping (Fig. S8). Nevertheless, *OxbZIP72^S71A^* lost the hypersensitivity to ABA with restored seed germination rates and seedling growth (Fig. 4c,d,f,h), indicating that SAPK10‐mediated phosphorylation on serine 71 is a key switch turning on the bZIP72 function in ABA signaling.

### Phosphorylated bZIP72 directly activates *AOC* transcription by binding to the G‐box in the promoter

**Fig. 5:**
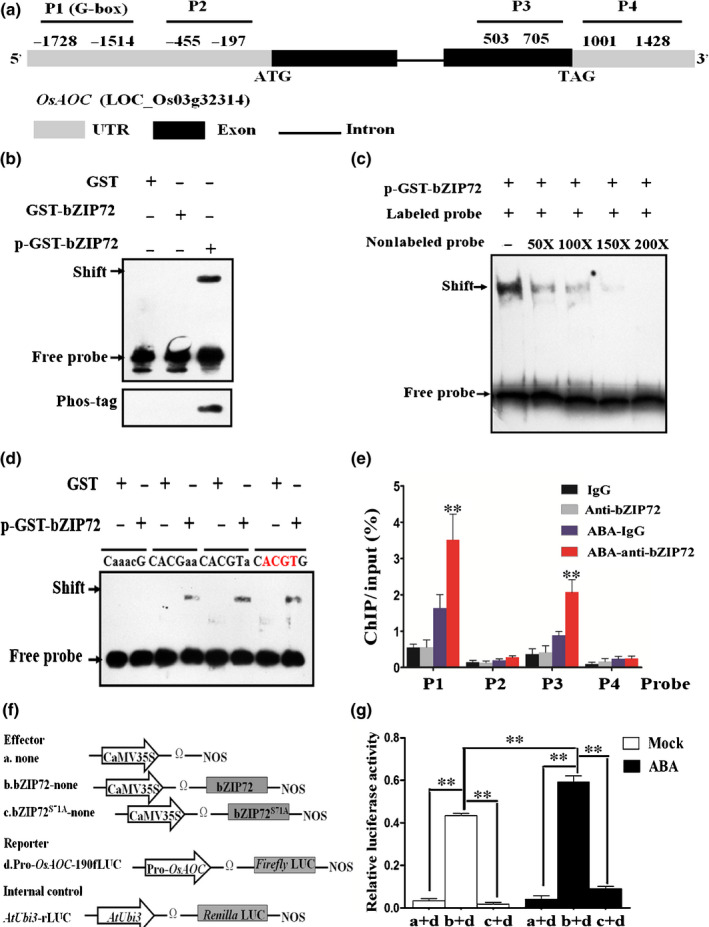
Phosphorylated bZIP72 directly activates *AOC* transcription by binding to the G‐box in the promoter. (a) Probe positions on *AOC* promoter and genome. Gray boxes, untranslated regions; black boxes, exons; black line, intron. Transcription starting site ATG was set as 0. Numbers indicate the distances (bps) of probes to the ATG. P1–P4, probes 1–4. (b) Electrophoresis mobility shift assay (EMSA) showed that p‐GST‐bZIP72 (phosphorylated GST‐bZIP72 band) binds with the P1 on the promoter of *AOC* in (a). Purified GST, GST‐bZIP72 and p‐GST‐bZIP72 were detected with anti‐GST antibody. p‐GST‐bZIP72 was detected with biotinylated Phos‐tag™ zinc BTL111 complex. GST, glutathione S‐transferase.(c) EMSA showed that p‐GST‐bZIP72 specifically bound to the G‐box in the P1 region of *AOC* promoter. The 50‐, 100‐, 150‐ and 200‐fold excess nonlabeled probes were applied as competitiors. (d) EMSA assay showed that CACGTG in the P1 region of *AOC* promoter was required for p‐GST‐bZIP72 to bind *AOC*. Probe 1 sequence (60 bp) contains G‐box (CACGTG) in (a), with the core element sequences ACGT in this motif marked by red letters. CACGTG was substituted by CaaacG, CACGaa and CACGTa in the mutant probe and the substitution nucleotide acids are marked in lowercase. (e) Chromatin immunoprecipitation‐quantitative PCR (ChIP‐qPCR) assay showed that abscisic acid (ABA) enhances bZIP72 binding to the promoter and coding sequence (CDS) regions of *AOC*. P1–P4 represents the regions shown in (a) detected by ChIP‐qPCR. The enrichment values were normalized to input. Immunoglobulin G (IgG) immunoprecipitated DNA was used as a control. Error bars indicate SD with biological triplicates. (f, g) Luciferase transient transcriptional activity assay in rice protoplast. Effectors: *35S:tNOS*, *35S:bZIP72:tNOS* and *35S:bZIP72^S71A^:tNOS*; Reporter: *proAOC:LUC*. Error bars represent means ± SD (*n* = 3) in (e) and (g). **, *P* < 0.01 by Student's *t*‐test analysis.

**Fig. 6:**
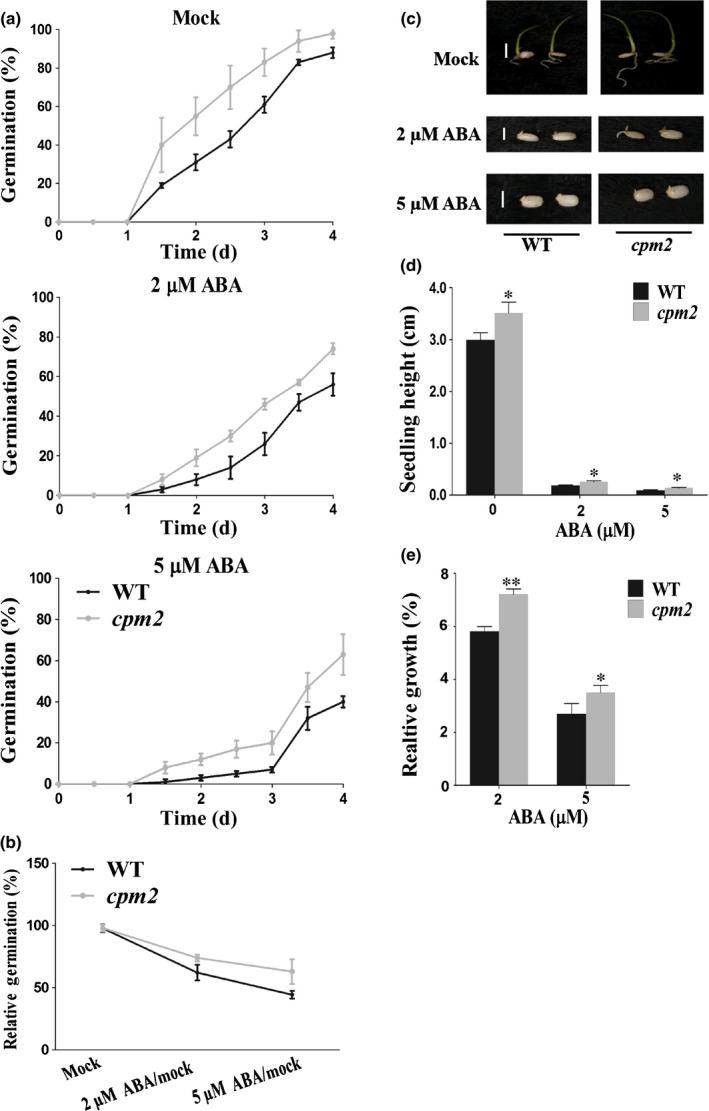
*cpm2*, a rice *AOC* mutant, displayed hyposensitivity to abscisic acid (ABA). (a) Germination time courses of the wild‐type (WT) and *cpm2* mutants grown on half‐strength Murashige & Skoog (½MS) medium containing different concentrations of ABA (0, 2, 5 μM). (b) The relative germination of the WT and *cpm2* seeds under ABA treatments were determined after 4 d and expressed as a percentage of those grown under the ‘mock’ condition. (c) Germination phenotypes of the WT and *cpm2* treated with 0, 2, 5 μM ABA. Photographs were taken on day 4. Bars, 1cm. (d) Seedling heights of the WT and *cpm2* in accordance with (c). (e) Growth inhibition of seedling height by ABA. The relative growth of the WT and *cpm2* seeds under ABA treatments were determined after 4 d and expressed as a percentage of those grown under the ‘mock’ condition. (a, d) Error bars indicate SD with biological triplicates (*n* = 3, each replicate containing 50 seeds) (a) and 50 biological replicates (*n* = 50) (d). Asterisks indicate the significance of differences between the WT and transgenic lines as determined by Student’s *t‐test* analysis in (d) and (e): *, *P* < 0.05; **, *P* < 0.01. *Oryza sativa* ssp.* japonica* cv Nihonmasari was used as the wild‐type.

As Gibberellins (GAs) and JA are actively involved in seed germination (Nambara *et al*., 2010), we examined the transcription level of some crucial GA or JA pathway genes in the germinating embryos of *OxbZIP72* and the WT. Intriguingly, a couple of JA biosynthesis step‐limiting genes, such as *LOX1*, *LOX2*, *AOS1*, *AOS2*, *AOC* and *OPR7* (Agrawal *et al*., 2003), were significantly upregulated in *OxbZIP72*, but remained unchanged in *OxbZIP72^S71A^*, while GA metabolism genes such as *CYP714B‐1* and *GA2ox6* displayed the opposite tendency (Fig. S9). As predicted by the online tool PlantCARE (Plant Cis‐Acting Regulatory Elements) (Lescot *et al*., 2002), promoters of *LOX1*, *AOS1* and *AOC* harbored G‐box *cis‐*elements, which are the conserved binding sites for bZIPs. Thus, we performed EMSA to test the DNA‐binding ability of bZIP72 on the promoter of these targets. As a result, no positive interactions were detected when unphosphorylated GST‐bZIP72 was used (Fig. S10). Nevertheless, we found that phosphorylated p‐GST‐bZIP72 could specifically bind to the probe 1 (P1) region of *AOC*, which is a master regulator of JA biosynthesis in the production of JA precursor 12‐oxo‐phytodienoic acid (12‐OPDA) (Fig. 5a,b). The EMSA binding was substantially weakened by nonlabeled, competitive probes in a dosage‐dependent manner (Fig. 5c). This result implied that the SAPK10‐mediated phosphorylation confers bZIP72 DNA‐binding ability to the promoter of *AOC*, in addition to enhancing bZIP72 protein stability. The protein‐DNA binding was compromised when some of the nucleotides in the conserved G‐box in the P1 region were mutated, demonstrating that the G‐box acts as a core site for recognition by bZIP72 (Fig. 5a,d). Subsequently, ChIP‐qPCR was performed to validate such binding *in vivo*. A total of four fragments representing the promoter, UTR and CDS regions were used for the examination (Fig. 5a). In support of the results of EMSA, bZIP72 was significantly enriched in the P1 region of the *AOC* promoter under ABA treatment, while there was no significant enrichment in the other fragments under the mock condition, except that the P3 region, which is located in the CDS region of *AOC*, exhibited slight bZIP72 enrichment (Fig. 5e). Finally, a dual‐luciferase (LUC) transient transcriptional activity assay was performed to determine the regulatory effect of bZIP72 on *AOC* transcription (Fig. 5f). In comparison with the empty effector, *pro35S:bZIP72:tNOS* drastically elevated the level of fLUC reporter, but such induction was blocked when the effector bZIP72 was replaced by bZIP72^S71A^, which is in accordance with the transcription pattern of *AOC* in *OxbZIP72* and *OxbZIP72^S71A^* transgenic lines, respectively (Fig. 5g). Notably, the upregulation of *AOC* by bZIP72 was significantly enhanced when exogenous ABA was applied (Fig. 5g), suggesting that ABA might induce SAPK10‐mediated phosphorylation on bZIP72 to promote the transcription level of *AOC*. Previous studies have clarified that a rice *AOC* mutant *cpm2* has lower endogenous JA concentration and unchanged ABA contents when compared with the WT (*O. sativa* L. ssp.* japonica* cv Nihonmasari) (Hazman *et al*., 2015). Seed germination assay revealed that *cpm2* also displayed reduced sensitivity to ABA, because the relative germination rates of *cpm2* of 2 μM/mock and 5 μM/mock were 76% and 64%, respectively, whereas these of the WT were 64% and 45% (Fig. 6a,b). *cpm2* also showed a significantly higher relative seedling growth than the WT (Fig. 6c–e). Taken together, these experiments strongly supported the possibility that *AOC* positively participates in ABA‐mediated seed germination and postgermination growth processes.

Additionally, we tested the binding ability of GST‐bZIP72 on the promoters of some rice GA pathway genes such as *GA3ox1*, *GA2ox5* and *GA20ox2* with G‐box motifs (Itoh *et al*., 2001; Spielmeyer *et al*., 2002; Lo *et al*., 2008), and found that GST‐bZIP72 was able to bind to the promoter of *GA20ox2*, indicating the involvement of bZIP72 in GA homeostasis as well (Fig. S10).

### ABA partially inhibits seed germination by inducing JA accumulation

**Fig. 7:**
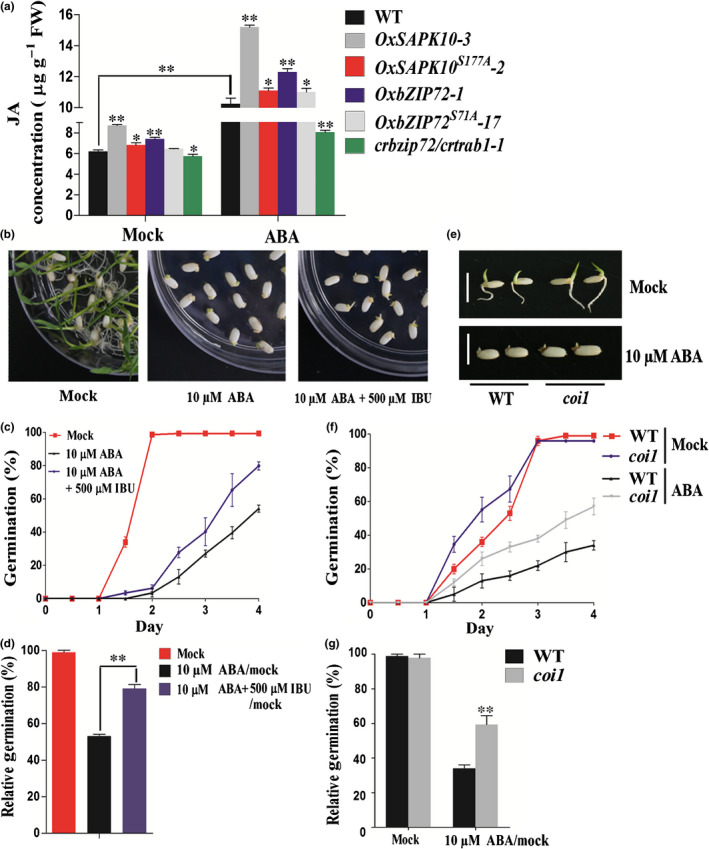
Abscisic acid (ABA) inhibits seed germination by partially inducing jasmonic acid (JA) accumulation in Nipponbare. (a) Measurement of JA concentrations in the seeds of the wild‐type (WT), *OxSAPK10*, *OxSAPK10^S177A^*, *OxbZIP72*, *OxbZIP72^S71A^* and *crbzip72/crtrab1* after being grown on half‐strength Murashige & Skoog (½MS) medium under mock or 10 μM ABA treatment for 4 d. Error bars indicate SD with three biological replicates (*n* = 3). Asterisks indicate the significance of differences between the WT and transgenic lines as determined by Student's *t*‐test analysis: *, *P* < 0.05; **, *P* < 0.01. (b) Germination phenotypes of the WT under mock, 10 μM ABA or 10 μM ABA + 500 μM Ibuprofen (IBU) treatment. Photographs were taken on day 10. (c) Germination time courses of the WT grown on ½MS medium under mock, 10 μM ABA or 10 μM ABA + 500 μM IBU treatment, respectively. (d) The relative germination of the WT seeds under 10 μM ABA or 10 μM ABA + 500 μM IBU treatment were determined after 4 d and expressed as a percentage of those grown under the ‘mock’ condition. (e) Germination phenotypes of the WT and *coi1* mutants under mock or 10 μM ABA treatment, respectively. Photographs were taken on day 4. Bars, 1 cm. (f) Germination time courses of WT and *coi1* grown on ½MS medium containing 0 or 10 μM ABA. (g) The relative germination of the WT and *coi1* seeds under 10 μM ABA treatment was determined after 4 d and expressed as a percentage of those grown under the ‘mock’ condition. (c, f) Error bars indicate SD with biological triplicates (*n* = 3, each replicates containing 50 seeds).

The ‘SAPK10‐bZIP72‐*AOC*’ regulatory pathway identified in the previous section hinted a positive regulatory pathway in the interplay between ABA and JA during seed germination. To address this issue, exogenous ABA was applied to germinating seeds of the WT. The JA accumulation level was significantly elevated when the WT germinating embryos were treated by ABA (Fig. 7a). Consistent with this result, a set of ABA signaling genes and JA pathway genes, including *AOC*, were drastically upregulated (Fig. S11). As both ABA and JA have been known as inhibitors for seed germination (Liu *et al*., 2015; Shu *et al*., 2016), it is likely that the final inhibition effects of ABA on seed germination may be partially based on the ABA‐promoted JA accumulation.

By blocking lipoxygenase, which is the key enzyme catalyzing the first step of JA biosynthesis, Ibuprofen (IBU) has been widely used as a JA inhibitor (Vick & Zimmerman, 1984; Staswick *et al*., 1991; Schaller & Stintzi, 2009). We found that the seed germination and post‐germination growth restricted by ABA were obviously relieved when IBU was applied (Figs 7b–d, S12). Additionally, *coi1*, a JA receptor mutant in rice (Yang *et al*., 2012), also showed reduced sensitivity to ABA in germination, as the relative 10 μM ABA/mock germination rate was 59% for *coi1* and only 34% for the WT. Seedling growth of *coi1* showed a similar reduced ABA sensitivity as the germination rate (Fig. 7e–g).

**Fig. 8:**
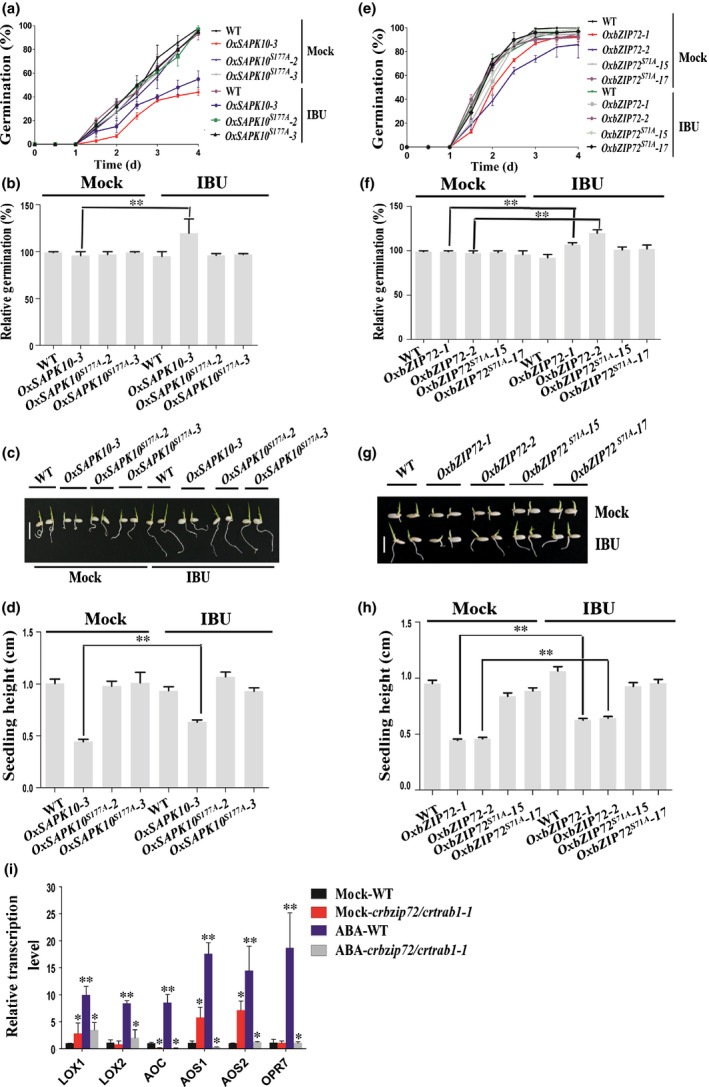
Ibuprofen (IBU) relieved the inhibition of *OxSAPK10* and *OxbZIP72* on seed germination in Nipponbare. (a) Germination time courses of the wild‐type (WT), *OxSAPK10* and *OxSAPK10^S177A^* grown on half‐strength Murashige & Skoog (½MS) medium under mock or 100 μM IBU treatment. (b) The relative germination of the WT, *OxSAPK10* and *OxSAPK10^S177A^* seeds under 100 μM IBU treatment were determined after 4 d and expressed as a percentage of those grown under ‘mock’ conditions. (c) Germination phenotypes of the WT, *OxSAPK10* and *OxSAPK10^S177A^* treated with 0 or 100 μM IBU. Photographs were taken on day 4. Bar , 1cm. (d) Seedling heights of the WT, *OxSAPK10* and *OxSAPK10^S177A^* in accordance with (c). (e) Germination time courses of the WT, *OxbZIP72* and *OxbZIP72^S71A^* grown on ½MS medium under mock or 100 μM IBU treatments. (f) The relative germination of the WT, *OxbZIP72* and *OxbZIP72^S71A^* seeds under 100 μM IBU treatment were determined after 3 d and expressed as a percentage of those grown under ‘mock’ conditions. (g) Germination phenotypes of the WT, *OxbZIP72* and *OxbZIP72^S71A^* treated with 0 or 100 μM IBU. Photographs were taken on day 4. Bar, 1cm. (h) Seedling heights of the WT, *OxbZIP72* and *OxbZIP72^S71A^* in accordance with (g). (i) Quantitative reverse transcription polymerase chain reaction (qRT‐PCR) analysis for transcript accumulation of abscisic acid (ABA) and jasmonic acid (JA) pathway genes in the seeds of the WT and *crbzip72/crtrab1*. Seeds were grown on mock or ½MS medium treated with 0 or 10 μM ABA for 6 h before harvest for total RNA isolation. (a, d, e, h, i) Error bars indicate SD with biological triplicates (*n* = 3, each replicate containing 50 seeds) (a, e), 50 biological replicates (*n* = 50) (d, h) and biological triplicates (*n* = 3) (i). (b, d, f, h) Asterisks indicate the significance of differences between the mock and IBU treatment (Student's *t*‐test analysis; **, *P* < 0.01). (i) Asterisks indicate the significance of differences between the WT and transgenic lines (Student's *t*‐test analysis; *, *P* < 0.05; **, *P* < 0.01).

The endogenous JA contents were also quantified in *OxSAPK10* and *OxbZIP72* germinating embryos with or without ABA treatment, respectively. Under nonABA conditions, the JA concentrations in *OxSAPK10* and *OxbZIP72* were significantly higher than the WT (Fig. 7a). However, the JA contents in *OxSAPK10^S177A^* and *OxbZIP72^S71A^* dropped drastically when compared with those in *OxSAPK10* and *OxbZIP72*, respectively (Fig. 7a). Moreover, the endogenous JA concentration in the *crbzip72/crtrab1* double mutant was significantly reduced under either mock or ABA treatment conditions, and a series of JA biosynthesis genes, including *AOC*, were downregulated in *crbzip72/crtrab1* (Figs 7a, 8i). Application of exogenous ABA showed similar inclinations as the nonABA conditions, but in a more severe manner (Fig. 8i). In accordance with the JA quantification assays, IBU relieved the inhibition on seed germination and seedling growth of *OxSAPK10* and *OxbZIP72*, while *OxSAPK10^S177A^* and *OxbZIP72^S71A^* showed no significant difference between the mock and IBU treatments, which is similar to what was seen in the WT (Figs 8a–h, S13). To this end, we concluded that ABA hinders seed germination partially through inducing JA accumulation.

## Discussion

### ‘SAPK10‐bZIP72‐*AOC*’ is a key pathway of ABA signaling in seed germination

The current study identified a key ABA signaling pathway, ‘SAPK10‐bZIP72‐*AOC*’, in rice seed germination. This pathway is supported by several layers of genetics and biochemical evidence: genetically, overexpression of *SAPK10* or *bZIP72* conferred plants with hypersensitivity to ABA during seed germination, whereas the double knockout of *bZIP72/TRAB1* or single knockout of *AOC* compromised the ABA sensitivity of seeds, suggesting that the three genes had similar positive functions in ABA signaling and seed germination; ABA‐inducible SAPK10 physically bound to and phosphorylated bZIP72, and the phosphorylation on bZIP72 could be enhanced by exogenous ABA; and SAPK10‐mediated phosphorylation strengthened the binding ability of bZIP72 on the *AOC* promoter and activated the transcription of *AOC*. Likewise, ABA also enhanced the bZIP72‐dependent activation on *AOC* transcription.

SAPK10 is the closest rice ortholog of OST1, which has been characterized as a key player in ABA signaling in *Arabidopsis* (Nakashima *et al*., 2009). Nevertheless, except for the discovery of its kinase activity on TRAB1 (Kobayashi *et al*., 2005), little has been known about the biological functions of SAPK10 until very recently. It is now clear that, together with SAPK8 and 9, SAPK10 could phosphorylate Tiller enhancer to block the GA signaling with elevated endogenous ABA concentration and retarded seedling growth, which is implicated in the ABA‐mediated inhibition of plant growth (Lin *et al*., 2015). SAPK10 is also involved in the ABA‐regulated rice flowering via phosphorylating bZIP77 in the FAC‐MADS15 pathway (D. Liu *et al*., 2019; X. Liu *et al*., 2019).

bZIPs are typical ABF TFs that can be phosphorylated by SnRK2 in ABA signaling (D. Liu *et al*., 2019; X. Liu *et al*., 2019). The current study showed that ABA‐inducible bZIP72 is subject to the protein phosphorylation regulation by SAPK10. *bZIP72* works redundantly with *TRAB1* to positively transmit ABA signal, as the ABA sensitivity in seed germination was attenuated only when both genes were mutated (Fig. 4a,b,e,g). Meanwhile, *OxbZIP72* became hypersensitive to ABA in seed germination (Fig. 4c,d,f,h), which is also consistent with its reported function in drought resistance (Lu *et al*., 2009). Interestingly, we found that *AOC* encoding an allene oxide cyclase in JA biosynthesis is directly transcribed by bZIP72. *AOC* has been implicated in coleoptile photomorphogenesis, abiotic stress resistance to salinity, as well as biotic stress resistance to piercing‐sucking insects and *Magnaporthe oryzae* (Riemann *et al*., 2013; Guo *et al*., 2014; Hazman *et al*., 2015). The ABA insensitivity of *cpm2* seeds hinted at an essential role of *AOC* in ABA signaling and crosstalk between ABA and JA in rice (Fig. 6).

### Comprehensive effects of SAPK10‐mediated phosphorylation on the ABA signaling elements

Protein phosphorylation represents a significant mechanism of the signal transduction of phytohormones such as ABA and brassinosteroids (BR) (W. Wang *et al*., 2014; Yang *et al*., 2017). The introduction or removal of a charged, hydrophilic phosphate group in the sidechain of amino acids could possibly change the structure of target protein by altering interactions with nearby amino acids, which finally imposes profound effects on the protein functions (Reinders & Sickmann, 2005). Under cold stress, phosphorylation facilitates the shuttling of 14‐3‐3 proteins from cytosol to the nucleus to interact with CBFs, while OST1‐dependent phosphorylation enhances the protein–protein interaction of BTF3 with their partners (Liu *et al*., 2017; Ding *et al*., 2018). Effects of phosphorylation on WRKYs are somehow elusive. For example, MPK3/MPK6 phosphorylates WRKY53 to enhance its transcriptional activity on defense‐related genes (Chujo *et al*., 2014). By contrast, WRKY72 had weakened trans‐activation on JA synthesis genes when phosphorylation occurred on its 129^th^ threonine (Hou *et al*., 2019). In this study, we revealed the comprehensive effects of SAPK10‐mediated phosphorylation on the target proteins. Biochemical evidence revealed that the autophosphorylation on Ser 177 of SAPK10 is crucial to maintaining its kinase activity on both itself and the substrate protein bZIP72, but this modification does not alter its protein–protein binding ability (Figs 1, 3, S1). Artificial blocking of the major autophosphorylation site Ser 177 of SAPK10 made the protein dysfunctional, because it was observed that overexpression of *SAPK10^S177A^* did not exhibit hyper sensitivity to ABA as the native SAPK10 did (Fig. 2). A similar phenomenon was observed on *OxbZIP72^S71A^*, the seed sensitivity to ABA of which remained at the same level as that of the WT (Fig. 4). However, the underlying molecular mechanisms were distinct; as indicated in the cell‐free protein degradation assay, constitutive phosphoryaltion on Ser 71 of bZIP72 made the protein more resistant to the degradation by 26S proteosome (Fig. 3f,g). Application of exogenous ABA achieved the same effect, and this is in agreement with the logic that ABA could enhance the SAPK10‐mediated phosphorylation on bZIP72 (Fig. 3). In addition to the protein stability, the phosphorylation strengthened the DNA‐binding ability of bZIP72 on the G‐box region of *AOC* promoter, and eventually elevated the transcriptional level of *AOC* (Fig. 5). Notably, despite the fact that we functionally characterized the major phosphosite on bZIP72, the minor phosphosites mediated by SAPK10 as well as phosphorylations catalyzed by other kinases are still in the dark. Identification and characterization of the other unknown phosphorylation events on bZIP72 will certainly be helpful to us in understanding the regulatory mechanism of bZIP72 in ABA signaling.

### ABA promotes JA accumulation to synergistically inhibit seed germination

Although their synergism relationship has been recognized for a long time, little is known about the molecular network between ABA and JA, especially in seed germination (Nambara *et al*., 2010). Recent work on *Arabidopsis* and wheat seed germination demonstrated that exogenous ABA elevated the endogenous JA concentration with the upregulation of a couple of JA biosynthesis genes (Ju *et al*., 2019). Unfortunately, because the research was more focused on how JA affected the ABA signaling and response, the possible mechanism of ABA‐regulated JA accumulation was untouched. Wang *et al* (2018) suggested that ABA may induce the expression of several plastid phospholipase such as *PLIP2* and *PLIP3* to promote the JA biosynthesis in chloroplast of *Arabidopsis* (Wang *et al*., 2018). In rice, the ABA‐responsive gene *OsbZIP82* positively regulates JA content, primarily through direct regulation of the JA metabolism genes, instead of biosynthesis genes (D. Liu *et al*., 2019; X. Liu *et al*., 2019). In this study, we linked the ABA signaling with JA production through a novel pathway, ‘SAPK10‐bZIP72‐*AOC*’, in which ABA promoted the JA accumulation in germinating seeds. Similarly, higher JA concentration was also detected in *OxSAPK10*, which has elevated endogenous ABA concentration as well as augmented ABA signaling. It appeared that a set of important JA biosynthesis genes, including *AOS1*, *AOS2* and *AOC*, were upregulated in the germinating embryos of *OxbZIP72* (Fig. S9). In particular, we found that *AOC* could be directly activated by bZIP72 after receiving the phosphorylation signal from the ABA signaling core element SAPK10 (Fig. 5). As a rate‐limiting gene of JA biosynthesis, the *AOC* mutant *cpm2* was found to be defective in producing JA precursor 12‐oxo‐phytodienoic acid (12‐OPDA), which ultimately led to lower JA concentration (Hazman *et al*., 2015). By conrast, overexpression of *AOC* resulted in greater JA accumulation in the plants (Guo *et al*., 2014). The reduced ABA sensitivity in *cpm2* suggested that AOC serves as a key node in the ABA signaling and ABA–JA interaction (Fig. 6).

Considering the inhibition effect of JA in seed germination (Wilen *et al*., 1991; Yang *et al*., 2012), it is rational to speculate that the final ABA‐imposed inhibition on seed germination might be the additive effects from both ABA and ABA‐induced JA. Indeed, this was attested by three independent germination assays in which the JA biosynthesis was blocked by using IBU or by knocking out *AOC*, or JA signaling was repressed by knocking down *COI1*. Under all the three conditions described, ABA sensitivities were significantly compromised (Figs 6, 7). Additionally, in *OxSAPK10* and *OxbZIP72* seeds, the inhibition caused by augmented ABA signaling could also be neutralized by IBU (Fig. 8). Therefore, we concluded that the ABA inhibition is partially based on the activated JA concentration.

As a few previous studies have shown that JA‐isoleucine (JA‐Ile), instead of JA, works as the active molecule triggering JA signaling (Thines *et al*., 2007; Katsir *et al*., 2008), it was concerned that the elevated endogenous JA concentration may not necessarily represent a higher JA response in case that JA‐JA‐Ile conversion is blocked. However, in the current study, the application of IBU relieved the ABA‐induced JA repression on *OxSAPK10* and *OxbZIP72* seeds (Fig. 8a,b,e,f), which means JA signaling is effectively working, and JA to JA‐Ile conversion should not be affected. Given the reported strong positive correlation between JA and JA‐Ile in plants (Suza & Staswick, 2008; Shimizu *et al*., 2013), we propose that JA‐Ile concentration should also be elevated with the increase of JA in this case. Additionally, JA can also be converted to 12‐OH‐JA without being converted to JA‐Ile, and the JA‐Ile peak might not be visible as it could be converted to its oxidated form quickly (Heitz *et al*., 2019). Hence the role of JA meta‐ and catabolism in seed germination of rice warrants further studies by using *jar1* mutant.

**Fig. 9:**
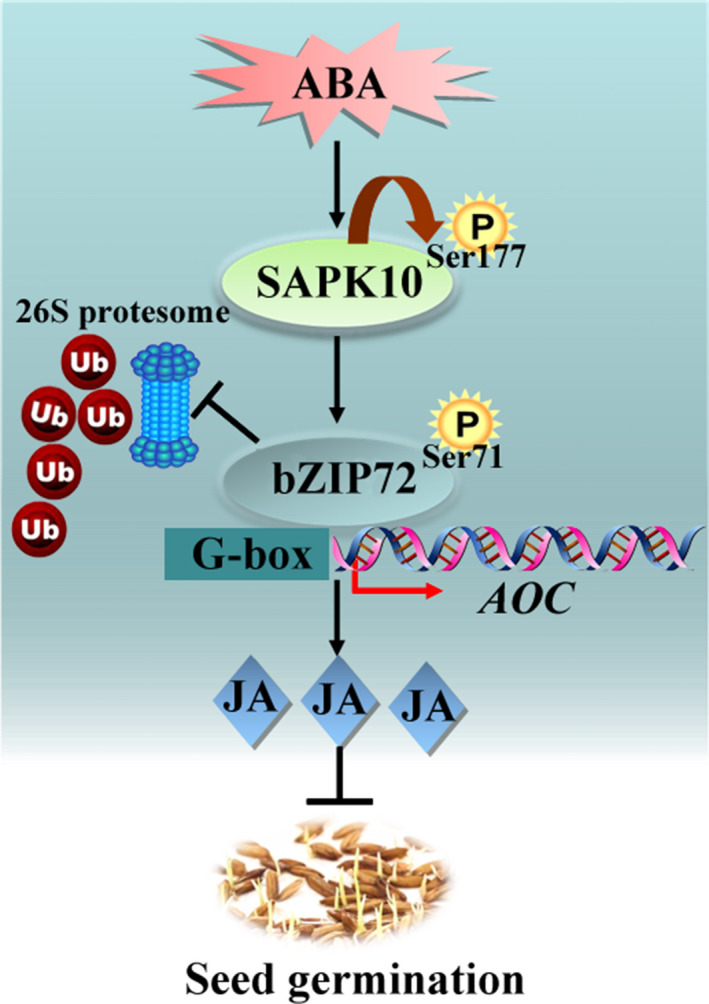
Working model for ‘SAPK10‐bZIP72‐*AOC*’ pathway in rice seed germination. P, phosphorylation group; Ub, ubiquitin; Ser, serine. Arrowheads show positive regulation, flat‐ended lines show negative regulation and the brown bent arrow represents phosphorylation.

A model summarizing the role of the ‘SAPK10‐bZIP72‐*AOC*’ pathway in rice seed germination is depicted in Fig. 9. Exogenous ABA confers autophosphorylation on the 177^th^ serine of SAPK10, which enables it to phosphorylate bZIP72 to a great extent on the 71^st^ serine (Fig. 9). The SAPK10‐dependent phosphorylation enhances bZIP72 protein stability against 26S proteasome‐mediated degradation as well as the DNA‐binding ability to the G‐box *cis*‐element of the *AOC* promoter, which elevated the *AOC* transcription, and finally increased the endogenous JA concentration to synergistically inhibit seed germination (Fig. 9).

## Author contributions

YW and JZ conceived and designed the experiments; YW, YH, JQ, HW, SW, LT and XT conducted the experiments; YW, YH, and JZ analyzed the data; and YW and JZ wrote the manuscript. YW and YH contributed equally to this work.

## Supporting information

Please note: Wiley Blackwell are not responsible for the content or functionality of any Supporting Information supplied by the authors. Any queries (other than missing material) should be directed to the *New Phytologist* Central Office.

**Fig. S1** Sequence alignment of T‐ loop region of 10 *Arabidopsis* and 10 rice SnRK2 family members.
**Fig. S2** Molecular characterization of *sapk10* mutant and seed germination in response to ABA.
**Fig. S3** qRT‐PCR analysis for transcript accumulation of nine SnRK2 family members (*SAPK1*‐*SAPK9*) in the seeds of WT or *crsapk10* lines, respectively.
**Fig. S4** Molecular characterization of *SAPK10*, *SAPK10^S177A ^*overexpressing transgenic lines.
**Fig. S5** Expression pattern of SAPK10 and bZIP72.
**Fig. S6** Molecular characterization of *crb*z*ip72*,* crtrab1* and *crb*z*ip72/crtrab1* mutants.
**Fig. S7** qRT‐PCR analysis for transcript accumulation of *TRAB1* in the seeds of *crbzip72* transgenic line.
**Fig. S8** Molecular characterization of *bZIP72* and *bZIP72^S71A ^*overexpressing transgenic lines.
**Fig. S9** qRT‐PCR analysis for transcript accumulation of JA and GA pathway genes in the seeds of the WT, *OxbZIP72 and OxbZIP72^S71A^* transgenic lines.
**Fig. S10** EMSA of bZIP72 on JA pathway genes *AOC*, *AOS1* and *LOX1* promoter regions and GA pathway genes *GA3ox1*, *GA2ox5* and *GA20ox2* promoter regions.
**Fig. S11** qRT‐PCR analysis for transcript accumulation of ABA and JA pathway genes in the seeds of the WT.
**Fig. S12** IBU relieved the inhibition of ABA on post‐germination growth.
**Fig. S13** SAPK10‐mediated retarded seed germination was relieved by exogenous application of IBU.

**Table S1** Sequences of primers used in this study.
