# Supplementary material for: Abscisic acid promotes jasmonic acid biosynthesis via a ‘SAPK10‐bZIP72‐AOC’ pathway to synergistically inhibit seed germination in rice (Oryza sativa)
Source: New Phytol. 2020 Jul 28;228(4):1336–53. doi: 10.1111/nph.16774 (PMC7689938; doi:10.1111/nph.16774)
Supplement: Supplementary file 1 — Fig. S1 Sequence alignment of T‐ loop region of 10 Arabidopsis and 10 rice SnRK2 family members. Fig. S2 Molecular characterization of sapk10 mutant and seed germination in response to ABA. Fig. S3 qRT‐PCR analysis for transcript accumulation of nine SnRK2 family members (SAPK1‐SAPK9) in the seeds of WT or crsapk10 lines, respectively. Fig. S4 Molecular characterization of SAPK10, SAPK10S177A overexpressing transgenic lines. Fig. S5 Expression pattern of SAPK10 and bZIP72. Fig. S6 Molecular characterization of crbzip72, crtrab1 and crbzip72/crtrab1 mutants. Fig. S7 qRT‐PCR analysis for transcript accumulation of TRAB1 in the seeds of crbzip72 transgenic line. Fig. S8 Molecular characterization of bZIP72 and bZIP72S71A overexpressing transgenic lines. Fig. S9 qRT‐PCR analysis for transcript accumulation of JA and GA pathway genes in the seeds of the WT, OxbZIP72 and OxbZIP72S71A transgenic lines. Fig. S10 EMSA of bZIP72 on JA pathway genes AOC, AOS1 and LOX1 promoter regions and GA pathway genes GA3ox1, GA2ox5 and GA20ox2 promoter regions. Fig. S11 qRT‐PCR analysis for transcript accumulation of ABA and JA pathway genes in the seeds of the WT. Fig. S12 IBU relieved the inhibition of ABA on post‐germination growth. Fig. S13 SAPK10‐mediated retarded seed germination was relieved by exogenous application of IBU. [file NPH-228-1336-s001.pdf]

New Phytologist

***New Phytologist* Supporting Information**

Article title: Abscisic acid promotes jasmonic acid biosynthesis *via* a “SAPK10-bZIP72-*AOC*” pathway to synergistically inhibit seed germination in rice (*Oryza sativa* L.)

Authors: Yifeng Wang, Yuxuan Hou, Jiehua Qiu, Huimei Wang, Shuang Wang[1], Liqun Tang, Xiaohong Tong, Jian Zhang

Article acceptance date: 15 June 2020

The following Supporting Information is available for this article:

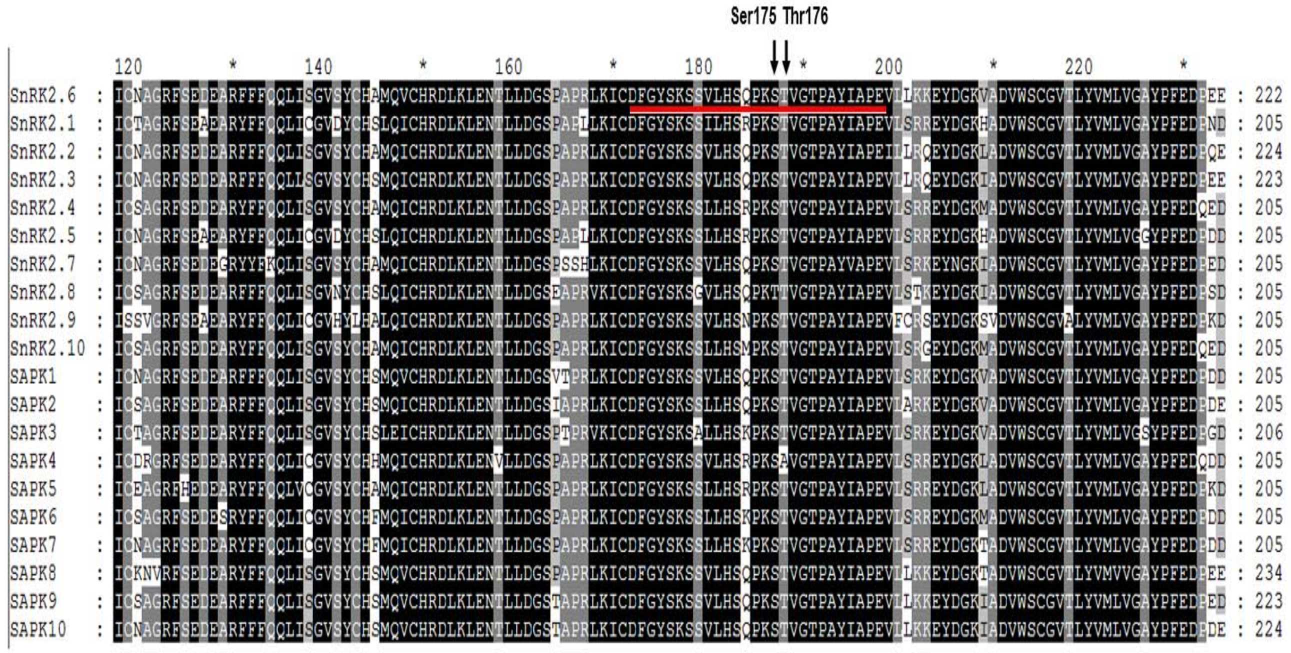

Fig. S1 Sequence alignment of T- loop region of 10 *Arabidopsis* and 10 Rice SnRK2 family members. Arrows indicate the residues of SnRK2.6 (OST1) phosphorylation sites. The putative T- loop predicted for SnRK2.6 is underlined in red.

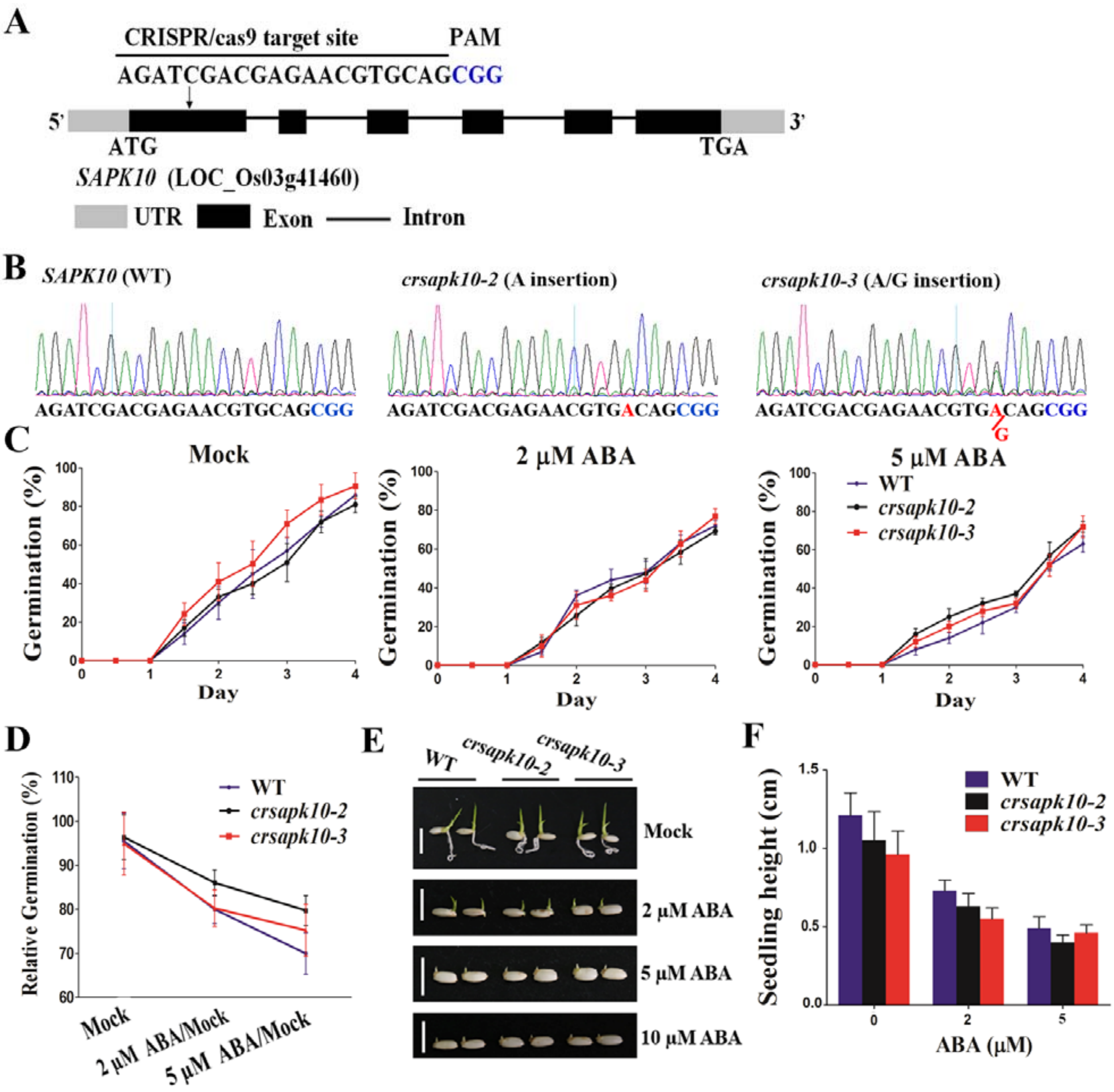

Fig. S2 Molecular characterization of *sapk10* mutant and seed germination in response to ABA. (A) Schematic presentation of the gene structure of *SAPK10* and CRISPR-cas9 editing site. Grey boxes: untranslated regions; Black boxes: exons; black line: intron. PAM: protospacer adjacent motif. (B) Sanger sequencing chromatograph of the CRISPR-cas9 target site in homozygous mutants of *crsapk10*. The letter in red represented the mutant sites. The letter in blue represented the PAM sequence. (C) Germination time courses of WT and *crsapk10* lines grown on half-strength MS medium containing different concentration of ABA (0, 2, 5 μM), respectively. (D) The relative germination of the WT and *crsapk10*

seeds under ABA treatments were determined after 4 days and expressed as a percentage of those grown on Mock condition. (E) Germination phenotypes of WT and *crsapk10* treated with 0, 2, 5 μM ABA, respectively. Photographs were taken on day 4. Bar=1cm. (F) Seedling heights of WT and *crsapk10* in accordance to (E). Error bars indicate SD with biological triplicates (n=3, each replicates containing 50 seeds) in (C) and 50 biological replicates (n=50) in (F).

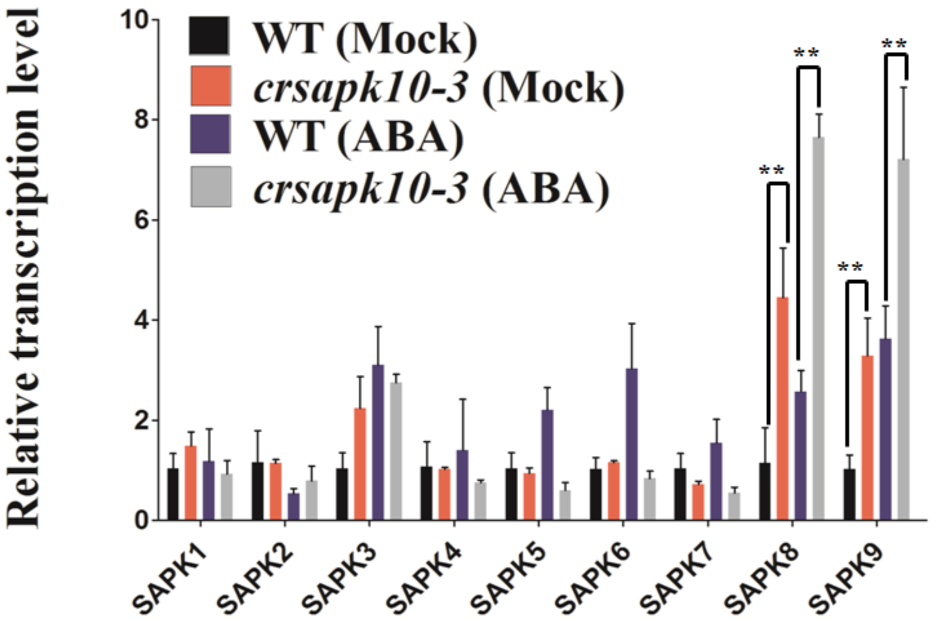

Fig. S3 qRT-PCR analysis for transcript accumulation of nine SnRK2 family members (*SAPK1*-*SAPK9*) in the seeds of WT or *crsapk10* lines, respectively. Seeds were grown on mock or 10 μM ABA treated half-strength MS medium for 6 hours before harvest for total RNA isolation. Error bars indicate SD with biological triplicates (n=3). Asterisks indicate the significance of differences between the WT and transgenic lines as determined by Student's *t-test* analysis: ** P<0.01.

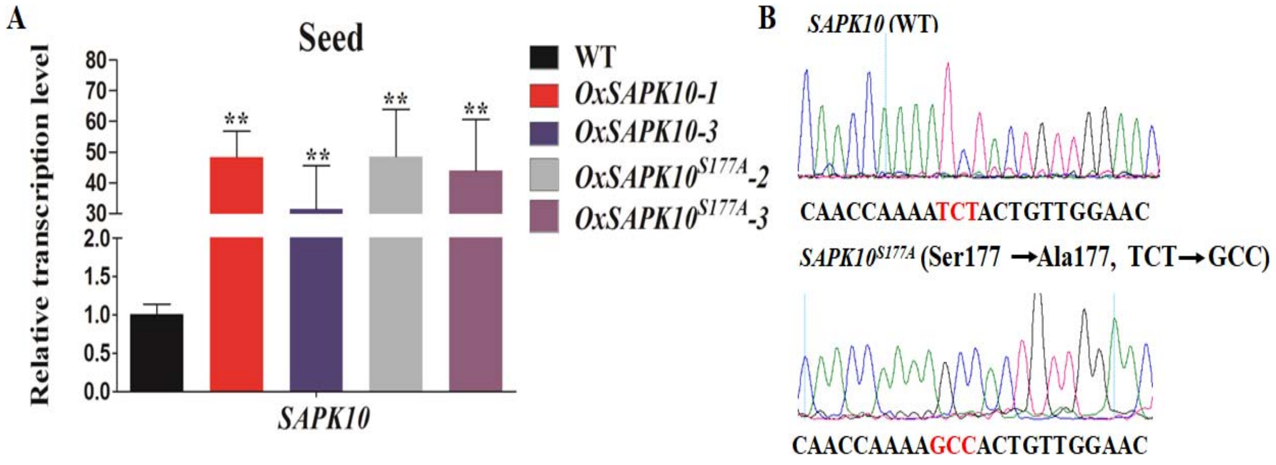

Fig. S4 Molecular characterization of *SAPK10*, *SAPK10*^{S177A} over-expressing transgenic lines. (A) qRT-PCR analysis for transcript accumulation of *SAK10* in the seeds of *OxSAPK10* or *OxSAPK10*^{S177A} transgenic lines, respectively. Seeds were grown on half-strength MS medium for 6 hours before harvest for total RNA isolation. Error bars indicate SD with biological triplicates (n=3). Asterisks indicate the significance of differences between the WT and transgenic lines as determined by Student's *t-test* analysis: ** P<0.01. (B) Sanger sequencing chromatograph of the mutated *SAPK10* cDNA in *OxSAPK10*^{S177A} transgenic lines. The letter in red represented the mutant sites.

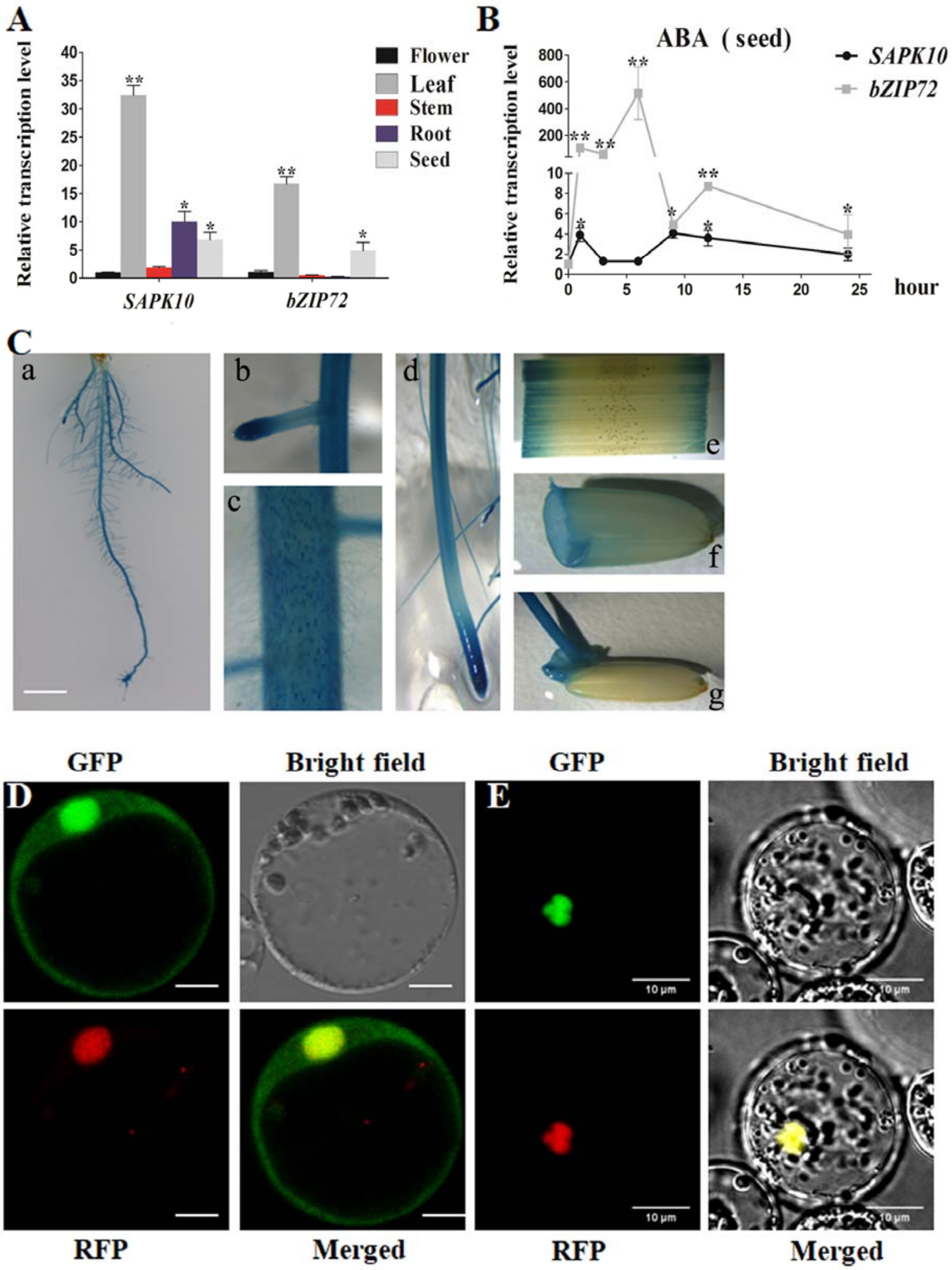

Fig. S5 Expression pattern of SAPK10 and bZIP72. (A) qRT-PCR analysis of *SAPK10* and *bZIP72* transcription level in flower, leaf, stem, root and seed, respectively. (B) qRT-PCR

analysis of *SAPK10* and *bZIP72* during seeds germination grown on half-strength MS medium treated with 10 μM ABA. (C) GUS staining of the primary root (a), lateral roots (b), root hairs (c), crown root (d), leaf (e), seed (f) and plumule (g) of the transgenic plants harboring the *proSAPK10*:*GUS* fused gene. Bar =1 cm in (a). (D and E) Subcellular localization of SAPK10 in (D) and bZIP72 in (E). *35S:SAPK10-GFP* or *35S:bZIP72-GFP* was co-transformed with a nuclear marker *35S:D53-mKate* (Zhou *et al*., 2016) into protoplast. Bar=10 μm. Error bars indicate SD with biological triplicates (n=3) in (A and B). Asterisks indicate the significance of differences between the WT and transgenic lines as determined by Student's *t-test* analysis: * $P<0.05$, ** $P<0.01$.

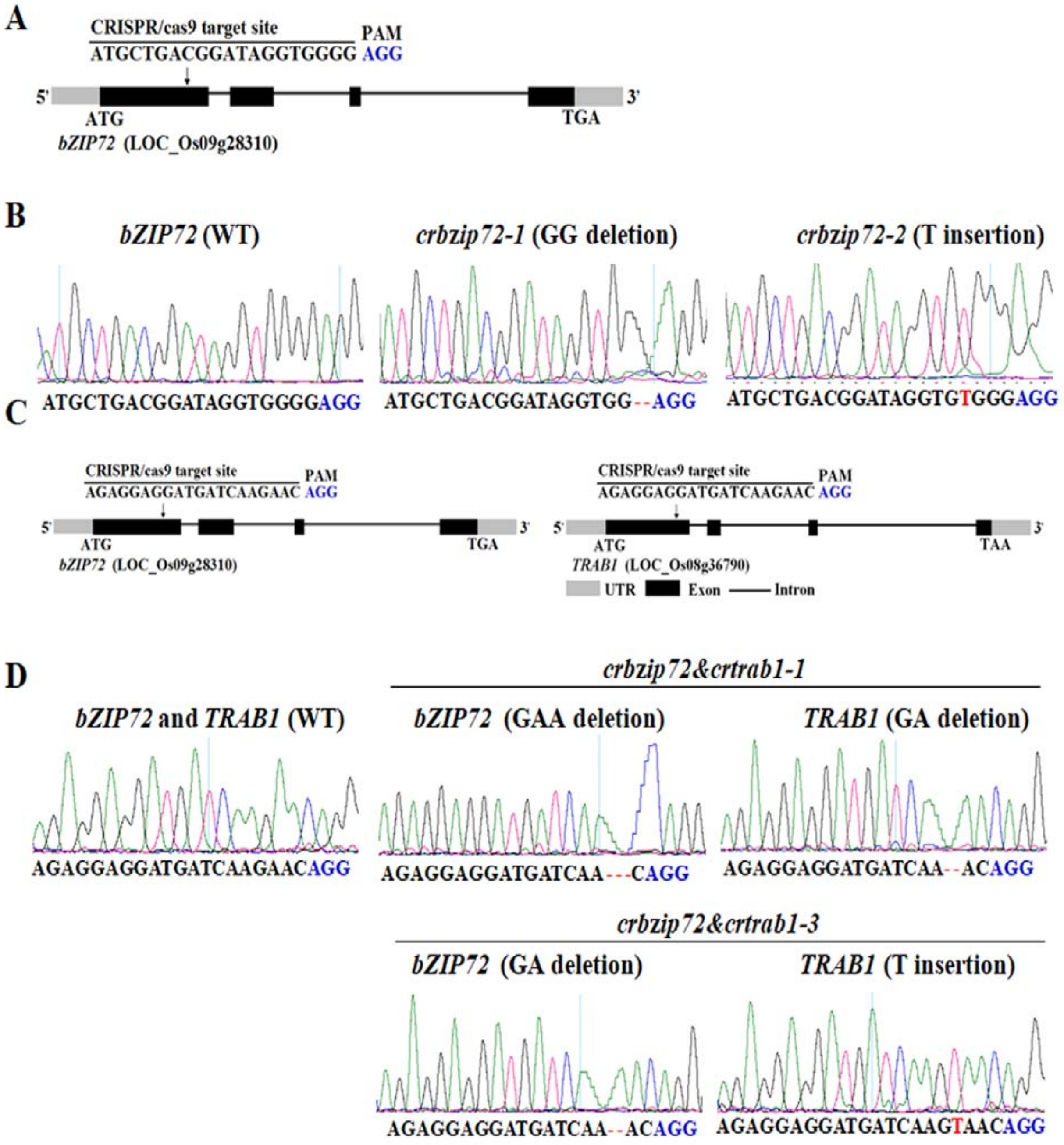

Fig. S6 Molecular characterization of *crbzip72*, *crtrab1* and *crbzip72/crtrab1* mutant. (A) Schematic presentation of the gene structure of *bZIP72* and CRISPR-cas9 editing site. PAM: protospacer adjacent motif. Grey boxes: untranslated regions; Black boxes: exons; black line: intron. (B) Sanger sequencing chromatograph of the CRISPR-cas9 target site in homozygous mutants of *crbzip72*. The letter in red represented the mutant sites. The letter in blue represented the PAM sequence. (C) Schematic presentation of the gene structure of *bZIP72* and *TRAB1.* Both genes shared the same CRISPR-cas9 editing PAM sequence. (D)

Sanger sequencing chromatograph of the CRISPR-cas9 target site in homozygous mutants of *crbzip72/crtrab1*.

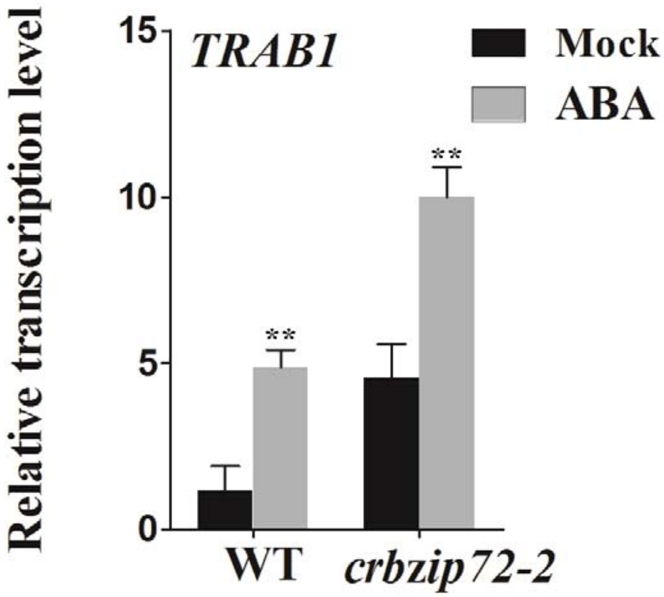

Fig. S7 qRT-PCR analysis for transcript accumulation of *TRAB1* in the seeds of *crbzip72* transgenic line. Seeds were grown on half-strength MS medium for 6 hours before harvest for total RNA isolation. Error bars indicate SD with biological triplicates (n=3). Asterisks indicate the significance of differences between the WT and transgenic lines as determined by Student's *t-test* analysis: ** P<0.01.

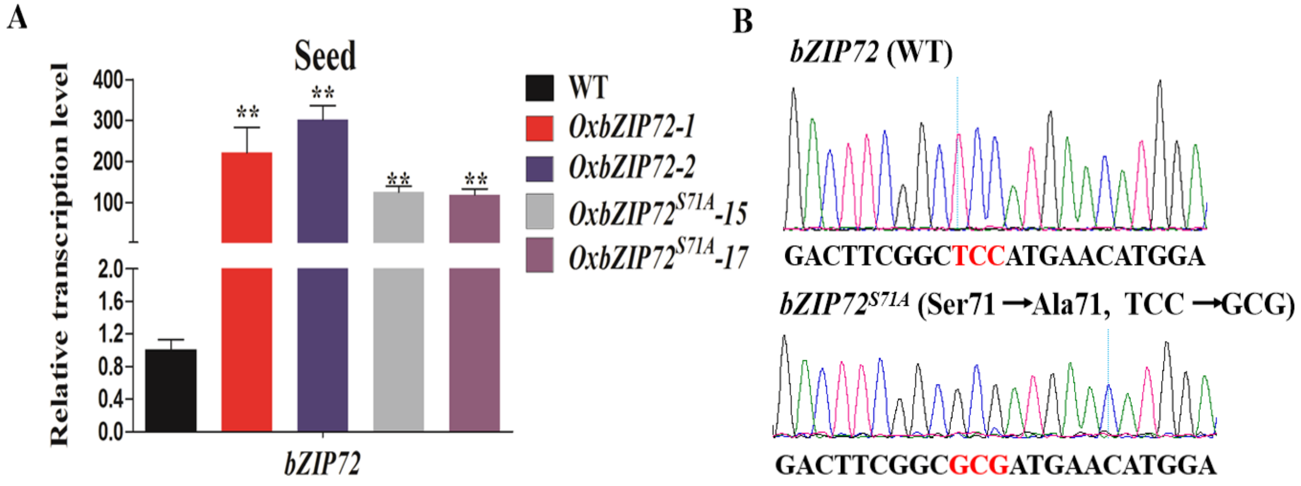

Fig. S8 Molecular characterization of *bZIP72* and *bZIP72*^{S71A} over-expressing transgenic lines. (A) qRT-PCR analysis for transcript accumulation of *bZIP72* in the seeds of *OxbZIP72 and OxbZIP72*^{S71A} transgenic lines, respectively. Seeds were grown on half-strength MS medium for 6 hours before harvest for total RNA isolation. Error bars indicate SD with biological triplicates (n=3). Asterisks indicate the significance of differences between the WT and transgenic lines as determined by Student's *t-test* analysis: ** P<0.01. (B) Sanger sequencing chromatograph of the mutated *bZIP72* cDNA in *OxbZIP72*^{S71A} transgenic lines. The letter in red represented the mutant sites.

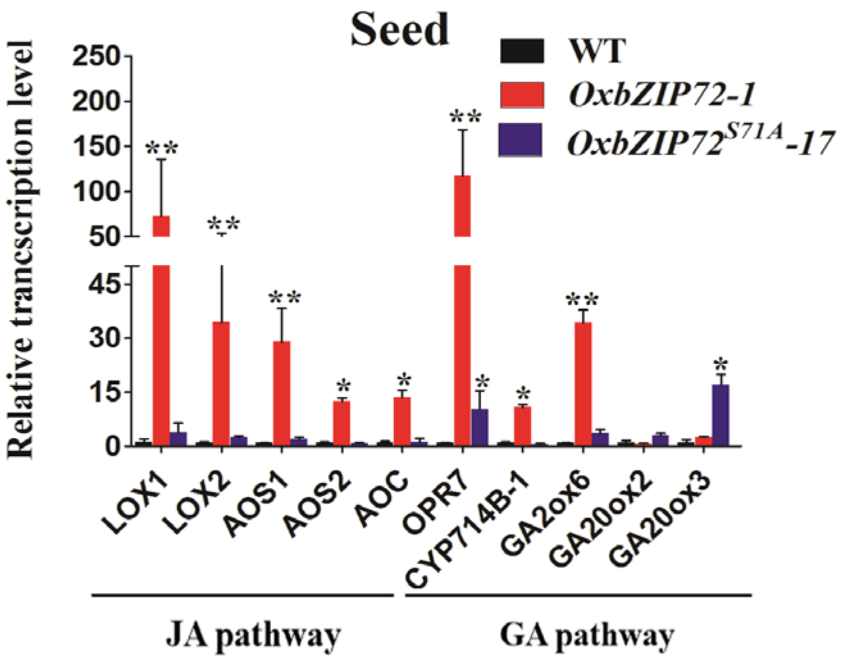

Fig. S9 qRT-PCR analysis for transcript accumulation of JA and GA pathway genes in the seeds of the WT, *OxbZIP72 and OxbZIP72*^{S71A} transgenic lines. Seeds were grown on half-strength MS medium for 6 hours before harvest for total RNA isolation. Error bars indicate SD with biological triplicates (n=3). Asterisks indicate the significance of differences between the WT and transgenic lines as determined by Student's *t-test* analysis: *P<0.05, ** P<0.01.

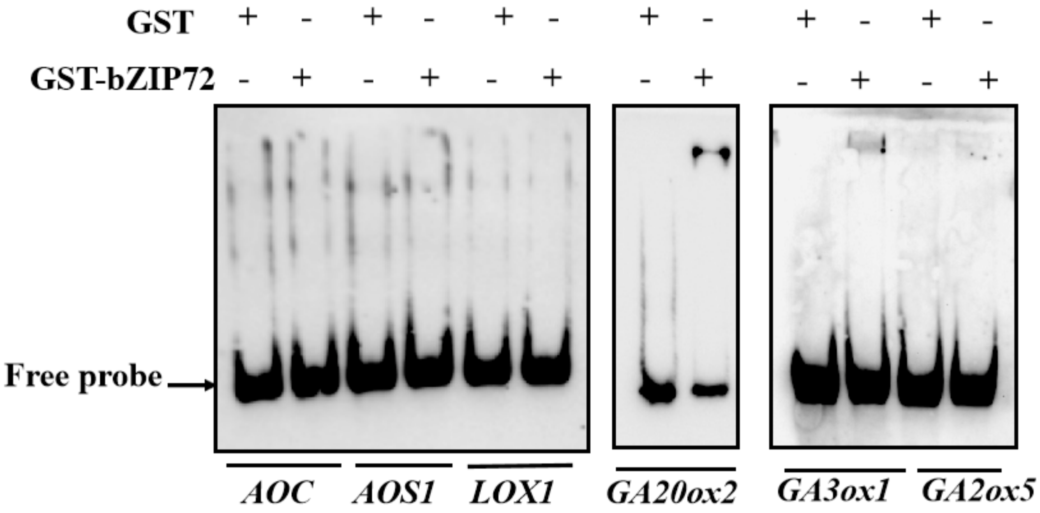

Fig. S10 EMSA of bZIP72 on JA pathway genes *AOC*, *AOS1* and *LOX1* promoter regions and GA pathway genes *GA3ox1*, *GA2ox5* and *GA20ox2* promoter regions.

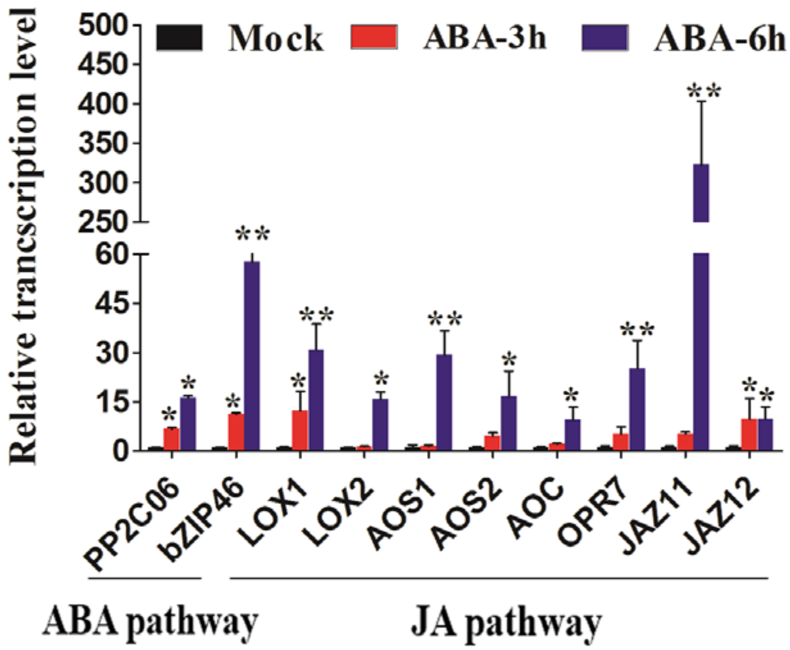

Fig. S11 qRT-PCR analysis for transcript accumulation of ABA and JA pathway genes in the seeds of the WT. Seeds were grown on half-strength MS medium treated with 0 or 10 μM ABA for 3 or 6 hours before harvest for total RNA isolation, respectively. Error bars indicate SD with biological triplicates (n=3). Asterisks indicate the significance of

differences between the mock-treated and ABA-treated seeds as determined by Student's *t-test* analysis: *P<0.05, ** P<0.01.

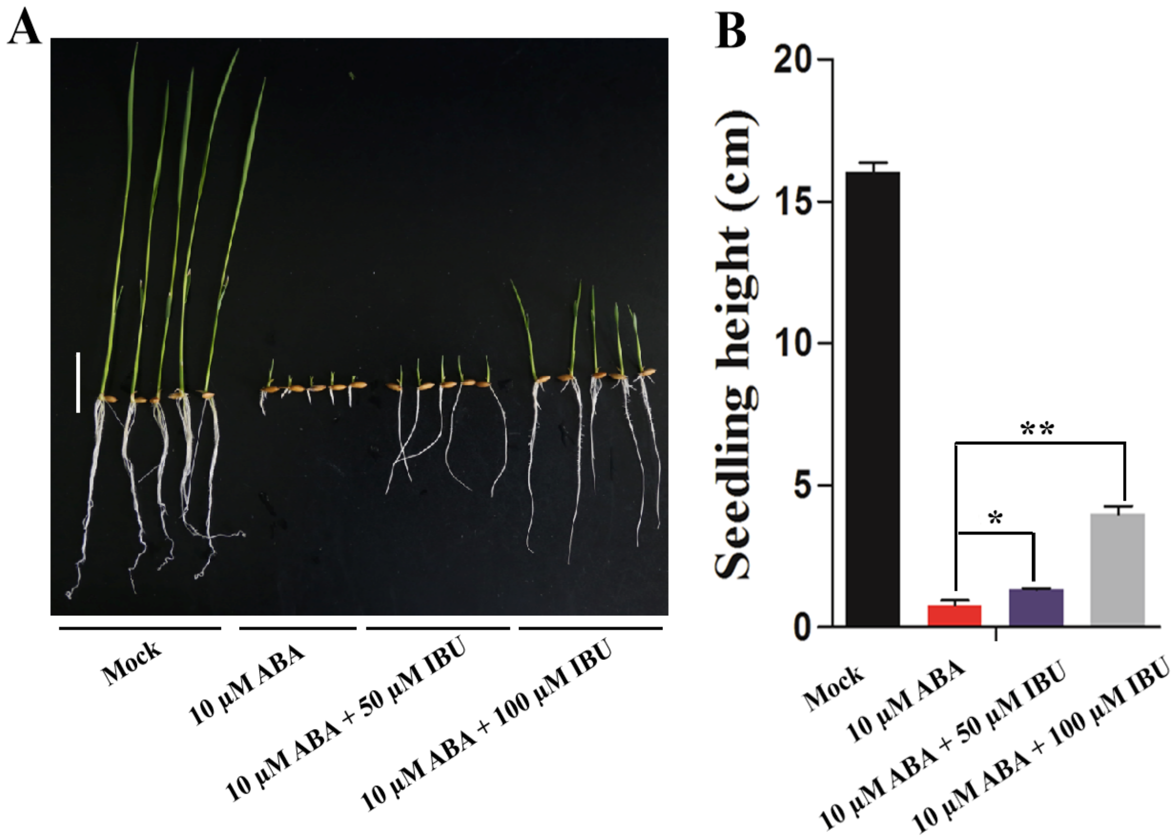

Fig. S12 IBU relieved the inhibition of ABA on post-germination growth. (A) Growth phenotypes of the WT after grown in nutrient solution containing 0, 10 μM ABA, 10 μM ABA + 50 μM IBU or 10 μM ABA + 100 μM IBU for 7 days, respectively. Bar= 2.5 cm. (B) Seedling heights of the WT in accordance to (A). IBU: Ibuprofen. Error bars indicate SD with 50 biological replicates (n=50). Asterisks indicate the significance of differences between the ABA-treated seedlings and ABA+ IBU treated seedlings as determined by Student's *t-test* analysis: *P<0.05, ** P<0.01.

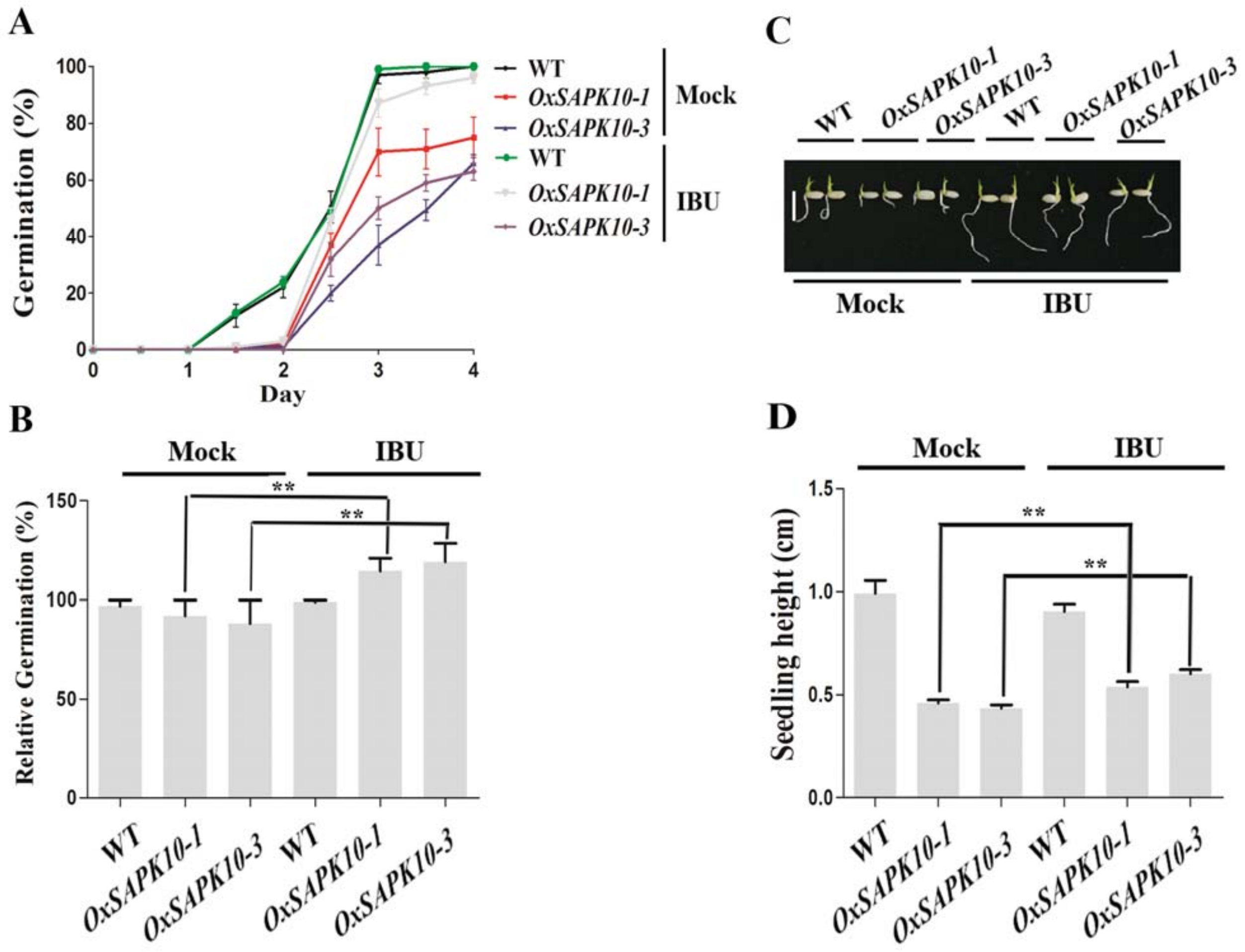

Fig. S13 SAPK10-mediated retarded seed germination was relieved by exogenous IBU applied. (A) Germination time courses of the WT and *OxSAPK10* grown on half-strength MS medium under mock or 100 μM IBU treatment, respectively. (B) The relative germination of the WT and *OxSAPK10* seeds under 100 μM IBU treatment was determined after 4 days and expressed as a percentage of those grown on Mock condition. (C) Germination phenotypes of the WT and *OxSAPK10* treated with 0 or 100 μM IBU, respectively. Photographs were taken on day 4. Bar=1cm. (D) Seedling heights of the WT and *OxSAPK10* in accordance to (C). Error bars indicate SD with biological triplicates (n=3, each replicates containing 50 seeds) in (A) and 50 biological replicates (n=50) in (D). Asterisks indicate the significance of differences between the mock and IBU treatment in (B and D) (Student's *t-test* analysis; ** P<0.01).
